# Drivers of Migration Readiness Among Romanian Pharmacy Students: An Extended Push–Pull–Mooring Model

**DOI:** 10.3390/healthcare14213745

**Published:** 2026-11-03

**Authors:** Pompei Mititean, Alin-Viorel Focșa, Maria Drăgan, Magdalena Bîrsan, Maria-Manuela Apostol, Ruxandra-Teodora Stan, Adina-Andreea Balea, Ioana-Cezara Caba, Cătălina Daniela Stan

**Affiliations:** 1 High School “Mircea cel Bătrân”, 012206 Bucharest, Romania; pompei.mititean@facultateademanagement.ro; 2 Faculty of Management, National School of Political and Administrative Studies, 012104 Bucharest, Romania; 3 Faculty of Pharmacy, Grigore T. Popa University of Medicine and Pharmacy Iasi, 700115 Iași, Romania; maria.wolszleger@umfiasi.ro (M.D.); magdalena.birsan@umfiasi.ro (M.B.); maria_manuela.apostol@umfiasi.ro (M.-M.A.); ruxandra-teodora.stan@umfiasi.ro (R.-T.S.); catalina.stan@umfiasi.ro (C.D.S.); 4 IOSUD-UTCN Doctoral School, Technical University of Cluj-Napoca, 400020 Cluj-Napoca, Romania; andreea.balea@campus.utcluj.ro

**Keywords:** pharmacy students, pharmaceutical workforce, healthcare workforce, workforce retention, health workforce planning, migration readiness, health policy, Push–Pull–Mooring, PLS-SEM

## Abstract

**Background/Objectives**: The potential migration of future pharmacists can affect healthcare systems’ ability to maintain a sufficient, stable, and adequately distributed workforce, with implications for the continuity of pharmaceutical services and the safe use of medicines. This study examines Migration Readiness (MR) among pharmacy students in Romania as a candidate aggregate indicator of future workforce-retention vulnerability and identifies the associated professional, economic, social, and structural factors. **Methods**: A quantitative, cross-sectional, and observational design was employed, based on 520 valid questionnaires. An extended Push–Pull–Mooring model was developed and tested, incorporating internal professional pressures (PUSH), professional prospects in Romania (DPO), the professional-economic appeal of external opportunities (PEP), the appeal associated with quality of life (QLP), personal and social barriers (PB), structural barriers (SB), and Migration Readiness (MR). The model was estimated using PLS-SEM in SmartPLS 4. The significance of relationships was assessed via bootstrapping with 10,000 subsamples, while predictive performance was evaluated using PLSpredict and CVPAT. **Results**: The model explains 39.2% of the variance in MR and demonstrates a good approximate fit (estimated SRMR = 0.038). PUSH is negatively associated with DPO (β = −0.387) and positively associated with PEP (β = 0.413) and QLP (β = 0.173). PEP is strongly associated with QLP (β = 0.523), while PEP and QLP are positively associated with MR (β = 0.272 and β = 0.221, respectively). DPO, PB and SB are negatively associated with MR (β = −0.128, β = −0.187 and β = −0.175, respectively). Significant specific indirect associations indicate that part of the association between professional pressures and MR operates through the assessment of domestic career prospects and external opportunities. **Conclusions**: Pharmacy students’ migration readiness reflects a multidimensional process in which internal professional conditions, the attractiveness of external opportunities, and mobility barriers act simultaneously. At an aggregate level, MR may represent a candidate perceptual indicator of future workforce-retention vulnerability; however, longitudinal validation against actual post-graduation employment and migration outcomes is required before operational workforce use. The results suggest that pharmaceutical workforce planning should combine measures addressing career prospects, working conditions, professional development, and quality of life, rather than focusing solely on remuneration.

## 1. Introduction

The migration of healthcare personnel represents a human resources management and policy issue, as it can reduce the availability of professionals, exacerbate geographical imbalances, and affect service continuity. From this perspective, mobility is not merely an individual career choice but a phenomenon with potential consequences for the health system’s capacity to retain the skills necessary for service delivery. The World Health Organization recommends strengthening the monitoring of professional mobility, retention, and distribution, and integrating this information into workforce planning mechanisms [1,2]. Recent reviews confirm that remuneration, working conditions, career opportunities, and quality of life remain key determinants of medical personnel’s intention to migrate [3,4,5].

The pharmaceutical workforce plays a significant role in this equation. Pharmacists contribute to the safe and effective use of medicines, patient counselling, therapy monitoring, access to treatment, and the provision of clinical and preventive services. In the Romanian community pharmacy setting, the relevance of these services is also reflected in evidence showing that patients’ satisfaction is closely linked to the perceived quality of pharmaceutical services and the overall pharmacy experience [6]. A reduction in the availability of this professional group can create pressure not only on community and hospital pharmacies but also on the medicines supply chain and the system’s capacity to expand patient-oriented pharmaceutical services. However, pharmacist migration has been studied less extensively than the mobility of physicians and nurses. Existing evidence links the intention to leave or migrate to remuneration, job satisfaction, professional stress, development opportunities, professional recognition, and work-life balance [7,8,9,10,11,12,13]. In Romania, both external mobility and dissatisfaction regarding job satisfaction, institutional support, and development prospects have been documented [14,15,16,17]. More recently, ref. [18] in a study of 542 practicing pharmacists in Romania, applied the Push-Pull-Mooring framework to analyse Migration Readiness and identified association between professional conditions, the importance attributed to migration benefits, relocation barriers, and willingness to engage in international mobility. The present study is not designed as a replication of that research. Rather, it addresses a different stage of pharmaceutical workforce pipeline and extend the PPM architecture conceptually. Whereas ref. [18] examined Migration Readiness among pharmacists already integrated into professional practice, the present study focuses on students before labour-market entry, when retention vulnerability concerns the conversion of educational output into domestic workforce supply. In addition, the current model distinguishes Domestic Professional Outlook (DPO) from professional push pressure, separates professional-economic from quality of life pull factors, differentiates personal-social from structural barriers, and tests specific and serial indirect pathways. Accordingly, the contribution lies in examining whether migration-related orientations are already identifiable before professional entry and whether these patterns may provide information relevant to future workforce retention. More broadly, the classical push-pull perspective on migration originates from Lee’s theory, which conceptualizes mobility as the result of interacting factors associated with the place of origin, the destination, intervening obstacles, and individual characteristics [19].

The Romanian context further illustrates why pharmaceutical workforce retention warrants attention. Romania had approximately 114 practicing pharmacists per 100,000 population in 2023, compared with an OECD average of 86 [20]. However, national supply does not necessarily translate into geographically balanced access to pharmaceutical services. In 2024, approximately 95% of the pharmacies, pharmaceutical points, and drugstores were located in urban areas and only 5% in rural areas, while approximately 54% of the Romanian population lives in rural areas [21]. Eurostat reported 1100 pharmacy graduates in Romania in 2023, indicating a substantial annual education output [22]. Comparable pharmacist-specific national vacancy data were not identified in the workforce sources used here, which limits vacancy-based assessment of unmet demand. Taken together, these indicators highlight the importance of examining not only the existing pharmacist stock but also the transition and retention of future professionals entering the domestic workforce.

From a workforce planning perspective, the migration of health professionals must be analyzed as a systemic issue rather than merely an individual choice. The World Health Organization emphasizes the pressures that staff shortages and unequal distribution exert on service access and continuity, and recommends the systematic monitoring of retention and mobility indicators [1,2]. For the pharmacy profession, such a perspective is relevant because the intentions of future pharmacists influence the capacity to provide medication-related services, counselling, and pharmacotherapy safety. In this context, MR is not treated as a definitive prediction of emigration or as a validated early-warning measure. Rather, it is examined as a candidate aggregate perceptual indicator of retention pressure within the future pharmaceutical workforce pipeline. Integrating such perceptual measures with longitudinal and administrative data on graduates, professional registration, geographical distribution, job placement and actual mobility could, if subsequently validated, provide earlier information on workforce vulnerability than the exclusive monitoring of the current stock of professionals [18].

The Push-Pull-Mooring (PPM) framework is well-suited to this issue because it distinguishes between factors driving an individual away from their current situation (push), the advantages offered by an external alternative (pull), and the anchors or obstacles that may limit change (mooring). The model is grounded in Lee’s migration theory [19] and the conceptualization of “moorings” proposed by Moon [23]; it has subsequently been used to explain switching and mobility behaviours [24,25,26,27,28,29]. In the context of workforce management, the advantage of the PPM framework is that it avoids the assumption that dissatisfaction automatically leads to departure; rather, the propensity to migrate stems from a comparison between internal conditions, external benefits, and the feasibility of relocation.

This study extends this logic in three directions relevant to the healthcare workforce. First, “pull” factors are categorized into professional-economic attraction (PEP) and quality-of-life attraction (QLP) to distinguish between incentives that can be addressed through professional policies and those related to the social environment and quality of life. Second, the domestic professional perspective (DPO) is introduced as a positive retention mechanism, distinct from the mere absence of “push” pressures. Research on organizational support and career development suggests that the visibility of internal opportunities can influence professional attachment and career intentions [30,31,32,33,34,35]. Thirdly, personal-social barriers (PB) and structural barriers (SB) are analysed separately, consistent with the distinction between migration aspiration and migration capacity [36,37,38].

The dependent variable is conceptualized as Migration Readiness (MR)—that is, multidimensional readiness for international mobility—reflected by the consideration of working abroad, the temporal proximity of a potential departure, perceived professional preparedness, and the assessment of migration as a realistic career solution. MR is not equivalent to actual migration and should not be used to classify individual students. Instead, at an aggregate level, it can be interpreted as a perceptual indicator of retention vulnerability within the future pharmaceutical workforce pipeline, complementing traditional indicators such as the number of graduates, geographic distribution, or job vacancies [2,37,38]. The primary objective of the research is to identify the mechanisms associated with pharmacy students’ willingness to migrate and to evaluate the relevance of these mechanisms for anticipating retention vulnerabilities and planning the future pharmaceutical workforce within the Romanian healthcare system. The study tests the relationships between PUSH, DPO, PEP, QLP, PB, SB, and MR, as well as the indirect and serial pathways through which professional pressures may translate into MR. Furthermore, the explanatory assessment is complemented by PLSpredict and CVPAT analyses to evaluate the model’s internal cross-validated predictive performance relative to incator-average and linear-model benchmarks. The intended contribution is therefore twofold: to extend PPM framework to the pre-professional stage of the pharmaceutical workforce pipeline and to examine whether Migration Readiness may serve as a candidate aggregate indicator for future retention vulnerability, subject to longitudinal and external validation.

## 2. Theoretical Framework and Hypothesis Development

Building on the conceptual background presented in Introduction, this section focuses on the theoretical relationship underlying the proposed model and the development of the study hypotheses. Research on healthcare professionals and students consistently identifies remuneration, working conditions, career prospects, training quality, quality of life, and social networks as relevant migration-related factors, although their relative importance varies across national and professional contexts [4,5,39,40,41,42]. Within pharmacy, available evidence similarly highlights career opportunities, remuneration, professional status, job satisfaction, and satisfaction with training or professional development [11,43,44,45,46]. The extended PPM framework adopted here distinguishes internal professional pressures and domestic career outlook from two forms of external attraction, professional-economic and quality-of-life related, and from personal-social and structural barriers to mobility. This distinction reflects evidence showing that favourable perceptions of external opportunities can coexist with family, financial, linguistic, cultural, or adaptation-related constrains [4,42,47]. Recent applications of the PPM framework to healthcare-worker mobility further support the simultaneous consideration of push, pull, alternative-evaluation, and mooring mechanisms without assuming that these dimensions operate with the same intensity or through the same pathways [28].

### 2.1. Internal Professional Pressures and Professional Outlook in Romania

The “push” construct captures the lack of professional prospects, job insecurity, and the absence of social recognition for the profession. These elements align with the factors of origin repeatedly identified in research on the migration of health human resources. A systematic review by ref. [5] shows that remuneration, working conditions, and career prospects frequently appear as determinants of migration, while an analysis by ref. [4], focusing on medical students, confirms the role of income, work culture, and professional advancement. In Ghana, ref. [40] demonstrate that push and pull factors are significant regarding physicians’ migration intentions, and ref. [39] identify remuneration, work-life balance, and working conditions as key reasons for British students’ interest in working abroad. In the field of pharmacy, ref. [11] find that negative career outlooks, lower incomes, and insufficient recognition are associated with migration intentions, while ref. [46] show that job satisfaction and work-life balance significantly impact pharmacists’ intentions to migrate. Among healthcare student populations, the perceived pressures are not exclusively professional; they can coexist with academic stress and anxiety—issues recently documented within the Romanian university context as well [48]. Crucially, PUSH and DPO are not conceptualized as opposite poles of the same continuum or as the same construct expressed through negatively versus positively worded items. PUSH captures perceived adverse characteristics of the domestic professional environment that students expect to enter, including limited career prospects, job insecurity, and insufficient professional recognition. DPO, by contrast, represent a broader and more forward-looking appraisal of whether Romania offers a credible and supportive career trajectory, including satisfaction with professional prospects, perceived appreciation of the profession, expectations regarding its future, and perceived support for young pharmacists. The distinction therefore concerns the referent and level of evaluation rather than item wording alone. A student may recognize specific weaknesses in the professional environment while still maintain relatively favourable overall assessment of a future career in Romania. Because the respondents are students rather than established employees, PUSH refers to perceptions of the professional environment rather than to accumulated personal employment experience. Based on this conceptual distinction, higher perceived professional pressure is expected to be associated with less favourable domestic professional outlook; we therefore formulate the following hypothesis:

**H1.**  *Internal professional pressures (PUSH) are negatively associated with career prospects in Romania (DPO).*

### 2.2. Formation of Professional and Economic Attractiveness

The professional and economic appeal of migration encompasses salary, working conditions, professional development, and access to technology and research. Available evidence consistently shows that these dimensions are central to the evaluation of opportunities abroad. In the review by ref. [4], income, the work environment, and professional advancement are among the factors most frequently cited by medical students. Ref. [39] identify remuneration and working conditions as key factors in the career decisions of British students, while ref. [41] highlight the quality of training and remuneration as drivers for Italian students considering opportunities abroad. Ref. [42] demonstrate that salary, work-life balance, and job security are integral to the motivational landscape of migration. Regarding pharmacists, [11] report the significance of external opportunities and higher salaries, while studies on pharmacy students’ aspirations indicate that salary, job opportunities, professional status, and the variety of career paths are relevant to future planning [43,44,45].

The literature thus supports both a contrast mechanism—whereby unfavourable domestic conditions make external alternatives more attractive—and a retention mechanism, through which favourable domestic professional prospects reduce the comparative advantage of the destination. However, most studies are cross-sectional and do not explicitly test the temporal order of these assessments; the relationships outlined below should be understood as structural associations compatible with the PPM, not as proven causal claims:

**H2.**  *Internal professional pressures (PUSH) are positively associated with external professional-economic attraction (PEP).*

**H3.**  *A more favourable professional outlook in Romania (DPO) is negatively associated with external professional-economic attraction (PEP).*

### 2.3. Formation of Attraction Associated with Quality of Life

Professional migration is linked not only to occupational earnings but also to the anticipation of a more favourable living environment. Ref. [4] categorize the determinants influencing medical students into domains that include the social environment and living conditions, while ref. [42] demonstrate that the desire for a better life, family well-being, and work-life balance are significant motivators. Ref. [5] identify lifestyle and family factors alongside economic and professional ones, and ref. [40] show that migration intention results from a combination of push and pull factors. Ref. [41] find that professional and personal motives combine to shape Italian students’ intentions to leave, while ref. [3] confirm the multidimensional nature of migration intentions among medical personnel.

The model distinguishes QLP from PEP to avoid treating all extrinsic benefits as a single construct. This separation allows for the testing of a more nuanced concept: internal professional pressures may enhance the perceived overall attractiveness of life abroad, whereas a favourable domestic outlook might mitigate this comparison. Furthermore, economic professional advantages can fuel the anticipation of a better quality of life. Existing literature frequently describes the coexistence of these dimensions but rarely explicitly tests the directional relationship PEP → QLP. Consequently, the hypotheses developed below represent a theoretical extension of current evidence and should be interpreted as such.

**H4.**  *Internal professional pressures (PUSH) are positively associated with the attraction associated with quality of life abroad (QLP).*

**H5.**  *A more favourable professional outlook in Romania (DPO) is negatively associated with the attraction associated with quality of life abroad (QLP).*

**H6.**  *External professional-economic attraction (PEP) is positively associated with the attraction associated with quality of life abroad (QLP).*

### 2.4. Determinants of Readiness for Migration

In this study, Migration Readiness (MR) is defined as a composite of a concrete orientation toward mobility: considering working abroad, the temporal proximity of a potential departure, perceived professional preparedness, and the assessment of migration as a realistic career solution. The choice of a concept broader than mere intention is justified by literature showing that mobility plans vary in their level of concreteness and that stated intention does not automatically translate into behaviour. Ref. [47] highlight the interplay between contexts and mechanisms in students’ decisions to leave, while ref. [3] emphasize the heterogeneity of intentions across countries. Refs. [42,49] also demonstrate that international exposure, external networks, and language skills correlate with the predisposition to migrate. This conceptualization builds upon the recent use of Migration Readiness in research on Romanian pharmacists, where the construct was treated as a pre-behavioural orientation involving the consideration of professional migration, its evaluation as a feasible career option, and the potential progression toward preparatory actions [18].

Evidence regarding professional pressures is robust: ref. [39] identify remuneration, work-life balance, and working conditions among the reasons for emigration, while ref. [5] show that economic and professional factors dominate in many contexts. Ref. [11] link the negative outlook on the profession and external opportunities to pharmacists’ migration intentions; ref. [40] highlight the contribution of push and pull factors, while ref. [46] show that, for pharmacists, work-life balance and job satisfaction are linked to migration intentions. At the same time, studies on students indicate that career prospects and the quality of training can act as retention factors [39,41,43]. Consequently, the following set of hypotheses is developed below:

**H7.**  *Internal professional pressures (PUSH) are positively associated with Migration Readiness (MR).*

**H8.**  *A more favourable professional outlook in Romania (DPO) is negatively associated with Migration Readiness (MR).*

**H9.**  *External professional-economic attraction (PEP) is positively associated with Migration Readiness (MR).*

**H10.**  *Attraction associated with quality of life abroad (QLP) is positively associated with Migration Readiness.*

### 2.5. Personal, Social and Structural Barriers

The PPM framework is particularly useful because it does not treat destination attractiveness as sufficient for mobility. Personal and social barriers include issues of adaptation, proximity to family and friends, uncertainty regarding career stability abroad, and assessments of the geopolitical and economic context. Ref. [4] show that family, finances, and patriotism are frequent factors influencing the decision to stay, while ref. [42] identify family dependency, a sense of alignment, and language difficulties among the primary barriers. Ref. [47] emphasize the role of social support and career certainty, and [5] demonstrate that family ties and personal circumstances can counterbalance professional pressures. Ref. [41] highlight bureaucratic obstacles, while ref. [50] document difficulties regarding cultural adaptation, communication, and the integration of migrant personnel. Structural barriers are addressed separately and include professional language proficiency, relocation costs, and administrative procedures. This distinction is justified because such barriers do not necessarily reflect personal attachment, but rather the practical capacity to translate intention into action. In recent literature, language, procedures, migration-related information, and financial resources emerge as either feasibility factors or obstacles, though their importance varies across systems and levels of preparedness [4,28,41,42,49]. The relevance of “mooring” factors is further supported by recent evidence regarding Romanian pharmacists, where relocation-related barriers showed a negative relationship with Migration Readiness, and family responsibilities and uncertainty regarding adaptation played a significant role in the formative composite of barriers [18]. Consequently, the following two hypotheses are proposed:

**H11.**  *Personal and social barriers (PB) are negatively associated with Migration Readiness (MR).*

**H12.**  *Structural barriers (SB) are negatively associated with Migration Readiness (MR).*

### 2.6. Mediation Mechanisms

A key contribution of the model is the testing of indirect mechanisms. Recent literature indicates that origin factors, destination benefits, and barriers tend to coexist; however, many studies report direct associations or regression models, while explicit serial chains are tested less frequently. The application of the PPM framework to medical personnel by [28] supports the integration of push, pull, and mooring factors, and the PLS-SEM study in ref. [46] demonstrates that, for pharmacists, the relationship between professional conditions and migration intention can be mediated by factors such as satisfaction. Reviews and studies involving students also suggest that dissatisfaction, external opportunities, quality of life, and feasibility are interdependent components of the same decision [4,5,39,40,42].

However, the precise order of the PEP → QLP → MR and PUSH → DPO/PEP/QLP → MR chains has not been consistently established in prior literature. The mediation hypotheses below are, therefore, deductive extensions of the PPM framework. In a cross-sectional design, confirming an indirect effect indicates that the data are consistent with the proposed mechanism, but it does not demonstrate temporal sequence or causality.

**H13.**  *QLP mediates a positive indirect association between PEP and MR.*

**H14a.**  *PEP mediates a positive indirect association between PUSH and MR.*

**H14b.**  *QLP mediates a positive indirect association between PUSH and MR.*

**H14c.**  *PEP and QLP serially mediate a positive indirect association between PUSH and MR.*

**H15a.**  *PEP mediates a negative indirect association between DPO and MR.*

**H15b.**  *QLP mediates a negative indirect association between DPO and MR.*

**H15c.**  *PEP and QLP serially mediate a negative indirect association between DPO and MR.*

**H16a.**  *DPO mediates a positive indirect association between PUSH and MR.*

**H16b.**  *DPO and PEP serially mediate a positive indirect association between PUSH and MR.*

**H16c.**  *DPO and QLP serially mediate a positive indirect association between PUSH and MR.*

**H16d.**  *DPO, PEP, and QLP serially mediate a positive indirect association between PUSH and MR.*

### 2.7. Critical Synthesis of the Literature and Positioning of the Model

Viewed as a whole, the literature on the mobility of health professionals supports an explanatory framework in which pressures in the country of origin, the attractiveness of the destination, and relocation obstacles are interdependent yet distinct. The classical foundations of this logic lie in ref. [19] push-pull theory, its extension via the concept of “moorings” proposed by ref. [23], and subsequent developments of the PPM framework regarding change-related behaviours [24,25]. More recent research confirms the framework’s robustness in contexts of mobility and changes but highlights that the intensity of these factors depends on the specific context, the type of profession, and perceived barriers [26,27,28,29]. Consequently, the present model does not treat PPM as a rigid scheme but rather as a framework capable of accommodating the distinctions between PUSH, DPO, PEP, QLP, PB, and SB factors.

Literature specific to the pharmacy profession points to a persistent combination of economic, organizational, and identity-related factors. The multicentre study by [7] demonstrated that migration can represent a significant form of pharmaceutical workforce loss, while research on stress and job satisfaction has linked organizational climate, role pressures, and satisfaction levels to the intention to leave the profession [8,51]. Reviews by ref. [9,10] show that pharmacist turnover is linked to satisfaction, development opportunities, management, workload, and recognition. Qualitative and mixed-methods studies from Australia and New Zealand further highlight the roles of limited autonomy, underutilization of skills, and career prospects [52,53], while more recent data confirm the importance of organizational support and advancement opportunities [13,32,33,34].

Regarding Romania, the literature offers consistent indications of retention vulnerabilities within the pharmacy profession. Ref. [16] highlighted determinants of hospital pharmacist satisfaction; ref. [14] analysed satisfaction, lifelong learning, and institutional support; and ref. [17] documented migration trends among Romanian pharmacists. More recent research on pharmacy graduates indicates links between satisfaction, perceived equity, and career commitment [35]. On a broader scale, an analysis by ref. [15] regarding the migration of Romanian medical personnel shows that the development level of the destination country and external professional networks influence mobility patterns. This evidence supports the simultaneous inclusion of internal pressures and external attractiveness, yet demonstrates that mere dissatisfaction is insufficient to explain migration readiness.

In a more recent PLS-SEM study, ref. [18] analysed migration readiness among practicing pharmacists in Romania using the PPM framework, showing that dissatisfaction with working conditions and remuneration is associated with the importance attached to migration benefits and with migration readiness, whereas “mooring” barriers are negatively associated with migration readiness. The present study extends this perspective to the pre-professional stage, examining similar mechanisms prior to students’ entry into the pharmaceutical workforce.

The dimension of professional retention must also be interpreted through organizational mechanisms. Support for career development can increase commitment and reduce the intention to leave [31], while perceived organizational support can act as an intermediary mechanism between professional experience and career intention [30]. Among students in health professions, professional identity, the learning environment, financial incentives, and career opportunities can shape migration intentions [54], and at the point of transition into the profession, perceptions of readiness and institutional support can influence career aspirations [55]. These results support distinguishing DPO from PUSH: the former construct captures the overall assessment of one’s professional future, whereas the latter captures more immediate negative pressures.

Another line of research indicates that migration intention must be differentiated from the actual capacity to migrate. The aspirations-capabilities framework proposed by ref. [37] and further developed by ref. [36], demonstrates that the aspiration for mobility and the ability to achieve it are distinct dimensions. Ref. [38] highlight the role of subjective and intangible factors in decision-making, while ref. [56] show that the professional integration of migrant medical personnel depends on language, adaptation, networks, and support. Studies involving physicians, nurses, and students confirm the role of family, finances, language, and the professional context in transforming mobility intentions into actual mobility [57,58,59,60]. This body of literature establishes the distinction between PB and SB and justifies the use of an MR construct that is closer to the concept of “readiness” than to a unidimensional intention.

From a workforce planning perspective, the migration of health professionals must be analysed as a systemic issue rather than merely an individual choice. The World Health Organization highlights the pressures that staff shortages and unequal distribution place on health systems and recommends the systematic monitoring of retention and mobility indicators [1,2]. In this context, Migration Readiness among pharmacy students may represent a candidate aggregate indicator of future workforce-retention vulnerability; however, its operational value requires longitudinal validation against actual post-graduation employment and migration outcomes, and it should not be interpreted as a predictor of individual migration [1,2,18,37,38].

Methodologically, recent literature supports the use of PLS-SEM when the objective combines explaining relationships between constructs with assessing predictive performance, and when the model includes both reflective and formative specifications. Recommendations regarding PLS-SEM reporting, construct evaluation, discriminant validity, and composite interpretation are detailed by ref. [61,62,63,64,65]. The risk of common method bias must be assessed with caution, without treating VIF as definitive evidence of the absence of bias ref. [66]. Predictive assessment must be distinguished from in-sample fit using PLSpredict and CVPAT [67,68,69]. These recommendations underpin the analytical strategy applied in the present study.

## 3. Materials and Methods

### 3.1. Research Design and Data Collection

This study aims to investigate the factors associated with the readiness for professional migration among pharmacy students in Romania and to assess the relevance of these factors for the retention and future workforce planning of the pharmaceutical profession. Drawing on the Push-Pull-Mooring theoretical framework, the proposed conceptual model analyzes the relationships between perceived professional pressures in Romania (PUSH), domestic professional prospects (DPO), the professional-economic appeal of external opportunities (PEP), the appeal of quality of life abroad (QLP), personal and social barriers (PB), structural barriers (SB), and Migration Readiness (MR). Unlike models that treat migration intention as the outcome of isolated factors, the structure of this article simultaneously examines the mechanisms through which perceived professional conditions in the home country influence the evaluation of external opportunities and readiness for mobility.

The questionnaire used in this study was developed based on the Push-Pull-Mooring framework and existing literature regarding the migration of healthcare professionals, the career intentions of medical and pharmacy students, workforce retention, and the transition from training to professional practice. The instrument is not a direct translation of a previously validated scale; rather, it conceptually integrates and adapts dimensions and items identified in other studies to reflect the specific characteristics of pharmacy students within the Romanian context.

The complete instrument included questions regarding informed consent, the respondents’ socio-demographic and academic characteristics, migration-related experience and general orientation, potential reasons for leaving, barriers associated with relocation, students’ perceptions of career prospects for pharmacists in Romania, preparation for mobility, and the assessment of external opportunities. Of the total questions in the questionnaire, 26 items were included in the final specification of the PLS-SEM model, distributed as follows: four items for PEP, three for QLP, three for PUSH, four for DPO, five for PB, three for SB, and four for MR. The remaining questions served descriptive, exploratory, or respondent-profiling purposes and were not included in the structural model.

The instrument development process aimed to ensure alignment between the definition of each construct and the content of the items used to measure it. The wording was adapted to be relevant to pharmacy students and understandable regardless of the respondents’ familiarity with the literature on professional migration. Items regarding professional-economic attraction were formulated based on literature concerning salaries, working conditions, professional development, and access to modern technologies and resources. Items concerning attraction associated with quality of life were grounded in studies highlighting standard of living, the social environment, and the functioning of the healthcare system in the assessment of external attractions. The PUSH and DPO indicators were developed based on literature regarding career prospects, the social recognition of the profession, job security, and support for future professionals; meanwhile, personal-social and structural barriers were constructed based on the PPM framework and studies concerning cultural adaptation, family relationships, relocation costs, language skills, and administrative procedures.

The content validity of the instrument was evaluated by a panel of seven experts: a psychologist, two associate professors, two full professors, a senior lecturer, and a teaching assistant. The experts analysed each construct and item in terms of relevance to the targeted conceptual domain, clarity of wording, appropriateness of terminology for the target population, and the extent to which the measured theoretical dimension was covered. The feedback provided was used to revise and clarify the items without altering the conceptual meaning of the constructs. Subsequently, the preliminary version of the questionnaire underwent cognitive pre-testing with a group of 25 pharmacy students to assess the comprehensibility of the questions, the suitability of response options, and the sequencing of items, as well as to identify any ambiguous or redundant wording. The responses obtained during the pre-testing phase were used exclusively to refine the instrument and were not included in the final analytical sample of 520 respondents. Following these two-evaluation stage-content validation and cognitive pre-testing—the final version of the questionnaire used for data collection in the main study was established.

**Figure 1:**
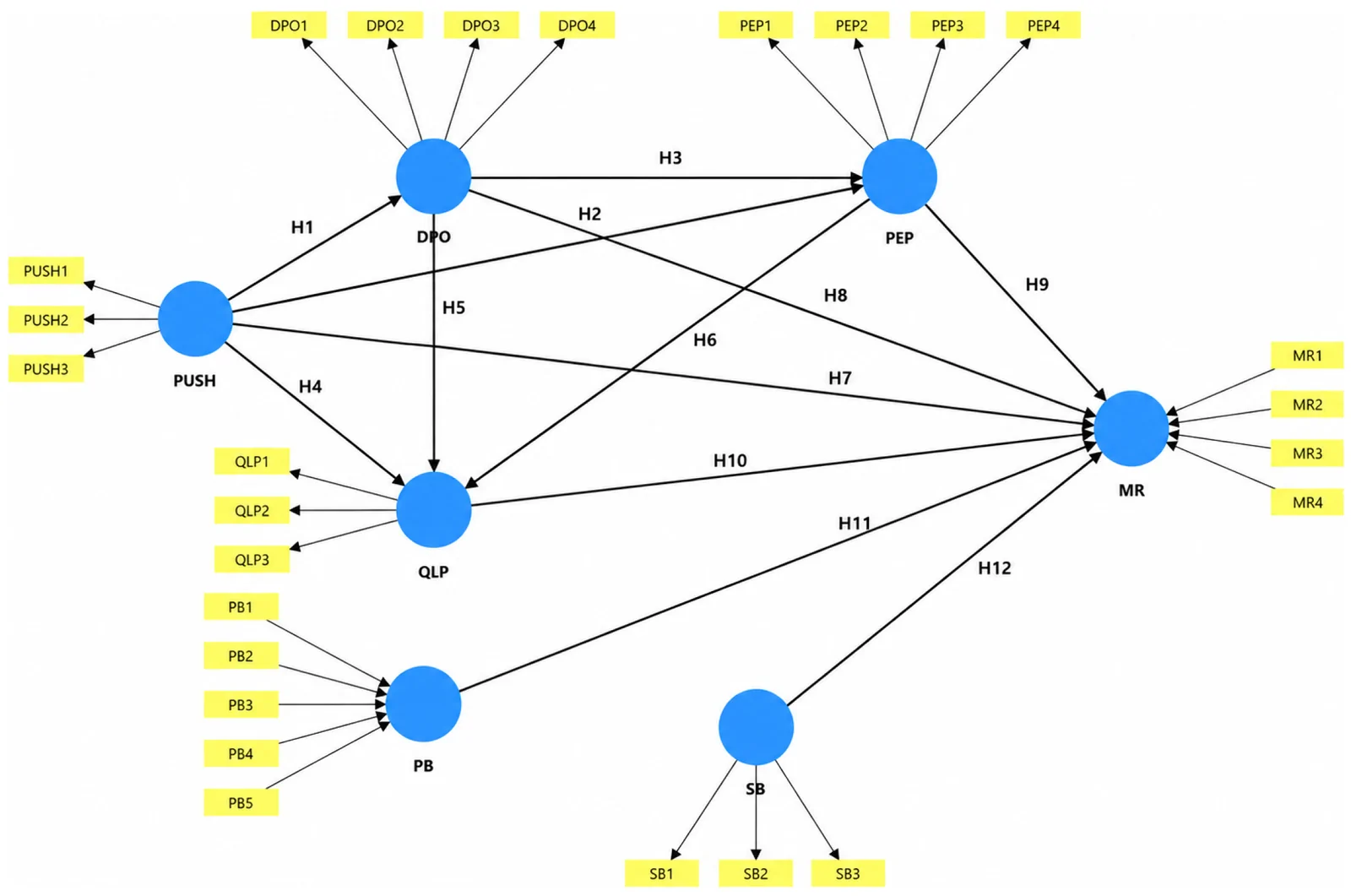
The proposed structural model.

The research employs a quantitative, cross-sectional, and observational design, with data collected via an online questionnaire created using Google Forms between March 9 and May 25, 2026. Online administration facilitated uniform data collection and access to students across various years of study. The data capture evaluations, perceptions, and orientations reported at the time of questionnaire completion; they do not derive from an experiment or a controlled intervention. Consequently, the relationships estimated in the proposed structural model are interpreted as compatible associations. The proposed structural model is presented in Figure 1.

Participation was voluntary, and inclusion in the analytical dataset was contingent upon prior informed consent. The target population comprises students currently enrolled in pharmacy degree programmes in Romania. Accordingly, the expression “pharmacy students in Romania” refers to enrolment in a Romanian pharmacy programme rather than to Romanian nationality, nationality was not used as an eligibility criterion. Eligibility was based on self-reported current enrolment in a pharmacy programme, provision of informed consent, and completion of the indicators required for structural model. Student status, year of study, and University/Faculty affiliation were self-reported and were not independently verified against institutional administrative records. A total of 524 questionnaire records were received. Four respondents did not provide informed consent and were excluded before analysis, resulting in a final analytical sample of 520 participants. No additional records were excluded from PLS-SEM analysis because complete data were available for all 26 indicators included in the final model.

The survey link was distributed via WhatsApp and email to faculty members teaching pharmacy students at other universities and was shared in Facebook groups for pharmacy students. Recruitment followed a non-probability convenience sampling strategy. Because the exact number of eligible students who received or viewed the invitation could not be determined, a conventional response rate could not be calculated. The distribution of respondents by university centre, based on the self-reported University/Faculty item, is presented in Table 1.

Sample adequacy was assessed considering both model complexity and statistical power and the analysis wis made with SmartPLS 4 [70]. MR is the endogenous construct with the largest number of direct predictors, being explained simultaneously by PUSH, DPO, PEP, QLP, PB, and SB. To complement the model-complexity criterion, a sensitivity power analysis was conducted using a multiple-regression approximation for the MR equation. With N = 520, α = 0.05, and six predictors, the sample provides 80% power to detect an overall effect of approximately f^2^ = 0.027 for the six-predictor equation [71]. This calculation concerns the joint explanatory contribution of the six predictors and should not be interpreted as the minimum detectable incremental effect of each individual structural path. Statistical power should also be distinguished from representativeness: the numerical size of the sample does not overcome the limitations of non-probability recruitment.

The sample is predominantly female (67.9% of respondents), and 66.7% of respondents come from urban areas. The majority are unmarried (90.4%), and 80.2% report having relatives or close friends who work or reside abroad. This last characteristic is relevant to the analysis of mobility because exposure through personal networks can reduce the uncertainty associated with a potential departure and increase familiarity with external opportunities, without, however, automatically implying an intention to migrate.

Approximately one-quarter of the respondents (26.0%) had prior international academic or professional experience. The distribution across years of study is relatively broad, with higher proportions in the final years; this allows for capturing the perceptions of students both at the beginning of their training and those nearing the transition to the labour market. The relatively high representation of final-year students is significant for the research objective, as the assessment of domestic and international professional opportunities tends to become more concrete as graduation approaches.

From the perspective of health workforce planning, the sample provides insights into the stage preceding entry into the pharmacy profession. The study does not aim to classify respondents as future migrants, nor does it seek to turn the research instrument into a tool for individual prediction. Instead, the analysis focuses on identifying—at an aggregate level—mechanisms and perceptual patterns that may signal retention vulnerabilities within the student-to-practicing-pharmacist pipeline. This distinction is crucial because, while a willingness to migrate may precede actual mobility, its translation into actual behaviour depends on subsequent opportunities, resources, and various family, professional, and institutional constraints.

### 3.2. Data Analysis Method

The proposed conceptual model was estimated using Partial Least Squares Structural Equation Modeling (PLS-SEM) with SmartPLS 4 [70]. The choice of PLS-SEM aligns with the research’s dual explanatory and predictive objective, the complexity of the structural model, and the inclusion of both reflective and formative constructs. The analysis strategy followed contemporary recommendations for PLS-SEM reporting, distinguishing between the assessment of the measurement model, the structural model, and predictive performance [61,62,63,64,65,72,73].

The measurement model was evaluated in the first stage. For the reflective constructs DPO, PEP, PUSH, QLP, and SB, the analysis covered outer loadings, Cronbach’s alpha, rho_A, composite reliability (rho_C), and average variance extracted (AVE). Regarding benchmarks, outer loadings of approximately 0.708 or higher were preferred; values exceeding 0.70 for internal consistency indicators were deemed adequate; and an AVE of at least 0.50 was used to support convergent validity. Indicators were not evaluated mechanically based solely on thresholds; decisions regarding their retention took into account both statistical contribution and the construct’s content validity.

Discriminant validity for the reflective constructs was assessed using the Fornell-Larcker criterion and the Heterotrait-Monotrait (HTMT) ratio. For the first criterion, the square root of the AVE for each construct was compared with its correlations with the other reflective constructs. HTMT was used as an additional primary criterion, given its higher sensitivity in detecting a lack of discriminant validity between constructs; values below the conservative threshold of 0.85 were considered indicative of discriminant validity [74].

Cronbach’s alpha, rho_C, and AVE were not used for the formative composites PB and MR, as the indicators represent distinct components contributing to the formation of the construct rather than interchangeable manifestations of the same latent variable. Evaluation focused on indicator collinearity via VIF, outer weights, outer loadings, and their significance as determined through bootstrapping. Since the instrument did not include a separate, redundant global indicator for PB and MR, the convergent validity of these composites could not be verified via redundancy analysis; this limitation is explicitly acknowledged in the interpretation of the measurement model.

On the other hand, the risk associated with relying on a single data collection method was addressed at both procedural and analytical levels. Participation was voluntary, and responses were gathered using an instrument structured into distinct sections, employing wording and response formats tailored to the content of the items. While these elements may partially mitigate artificial response consistency, they do not entirely eliminate common method variance. The subsequently reported VIF values are used to assess collinearity within the measurement and structural models; they are not interpreted as definitive proof of the absence of common method bias, in line with recommendations for methodological caution.

The structural model was evaluated in the second stage. Prior to interpreting path coefficients, the collinearity among the predictors of each endogenous construct was examined using Inner VIF. Subsequently, the analysis covered t-values, *p*-values, standardized path coefficients, bootstrap confidence intervals, coefficients of determination (R^2^), adjusted R^2^ values, and effect sizes (f^2^). The significance of direct and indirect relationships was tested using a bootstrapping procedure with 10,000 subsamples, a two-tailed test, and a 5% significance level. Confidence intervals were used alongside *p*-values to assess the robustness of the estimates.

Because perceived professional preparedness may represent capability without necessarily implying migration intention, a sensitivity analysis was conducted by re-estimating the complete structural model after excluding MR3 from the formative MR composite. The alternative specification retained MR1, MR2, and MR4, while all the other measurement specifications and structural relationships, estimation settings, and the analytical sample were unchanged.

Mediation analysis was conducted based on specific indirect effects rather than a sequence of separate tests for direct effects. Both simple mediation and the serial paths specified in the model were examined: PEP → QLP → MR; PUSH → PEP → MR; PUSH → QLP → MR; PUSH → PEP → QLP → MR; DPO → PEP → MR; DPO → QLP → MR; DPO → PEP → QLP → MR, as well as paths including PUSH → DPO preceding the mediators. An indirect effect was considered supported when the bootstrap estimate was significant and the confidence interval did not include zero. The classification of mediation took into account the sign and significance of the direct and indirect effects. Given the cross-sectional design, these classifications describe patterns of statistical association and do not establish temporal ordering or causal mediation.

Approximate model fit was described using SRMR, and NFI was reported separately for the saturated and estimated models; however, fit indices were not used as a substitute for evaluating reliability, validity, explanatory power, or predictive performance. In the PLS-SEM context, the model was evaluated using a combined approach, wherein approximate fit complements—rather than replaces—criteria specific to measurement and structural models.

In addition to in-sample explanatory power, predictive performance was assessed using PLSpredict with 10-fold cross-validation and 10 repetitions. For endogenous construct indicators, Q^2^_predict values and prediction errors (RMSE and MAE) were analyzed. PLS-SEM predictions were compared against a benchmark based on indicator means and a linear reference model, in accordance with the recommendations of ref. [67]. Predictive assessment was supplemented by the Cross-Validated Predictive Ability Test (CVPAT), which statistically tests differences in predictive loss between PLS-SEM and reference benchmarks [68,69]. A significant negative difference in loss indicates superior predictive performance for PLS-SEM. The simultaneous use of PLSpredict and CVPAT allows for distinguishing predictive relevance from mere explanatory power and mitigates the risk of interpreting a higher R^2^ as sufficient evidence of out-of-sample performance.

By combining the assessment of the measurement model, the structural model, indirect effects, and predictive performance, the analytical strategy aims not only to identify statistical associations between constructs but also to evaluate the model’s utility for the aggregate analysis of retention vulnerability. In this context, MR is not used for the individual classification of respondents, nor are predictive results interpreted as a definitive forecast of actual migration. Instead, the model provides a framework for identifying factors that, at a group level, may signal pressures on the future pharmaceutical workforce pipeline.

The structural model tested includes the relationships PUSH → DPO, PUSH → PEP, DPO → PEP, PUSH → QLP, DPO → QLP, PEP → QLP, PUSH → MR, DPO → MR, PEP → MR, QLP → MR, PB → MR, and SB → MR. Furthermore, simple and serial indirect effects were analyzed via DPO, PEP, and QLP to examine whether professional pressures and internal career prospects are associated with MR through changes in the attractiveness of external opportunities.

### 3.3. Measurement of Constructs

The indicators used in the research were defined based on the conceptual nature of the constructs analyzed. DPO, PEP, PUSH, QLP, and SB were operationalized as reflective models, as the indicators were conceived as correlated manifestations of an underlying assessment and—within each construct—were expected to vary in the same direction. In contrast, PB and MR were operationalized as formative models, as their components capture distinct, non-interchangeable dimensions that collectively contribute to the formation of the construct. The distinction between reflective and formative constructs was established conceptually prior to interpreting the statistical results, in accordance with recommendations regarding PLS-SEM construct specification [62,64].

The conceptual model comprises seven constructs. PUSH captures professional pressures perceived in Romania and is measured by the lack of career prospects, job insecurity, and the lack of social recognition for the profession. DPO captures the internal professional perspective through satisfaction with career prospects, the social appreciation of pharmacists, the assessment of the profession’s future, and the perception of support provided to young pharmacists. PUSH and DPO were specified as conceptually distinct constructs a priori. PUSH reflects perceived adverse characteristics of the professional environment, whereas DPO captures a broader prospective assessment of domestic career viability. Their distinction therefore concerns the referent and the level of evaluation rather than positive versus negative item wording alone. PEP reflects the professional and economic appeal of external opportunities, including salary, working conditions, professional development, and access to modern technology and research. QLP captures the broader dimension of the appeal of life abroad through cultural experience, quality of life, and the quality of the healthcare system.

The two categories of barriers were kept separate to avoid aggregating conceptually distinct mechanisms. PB is a formative construct comprising the fear of adapting to a new culture, the desire to remain close to family and friends, uncertainty regarding career stability abroad, and the geopolitical and economic contexts. These components can exist independently; a respondent reporting a high family-related barrier does not necessarily report a high geopolitical or economic barrier. SB was specified reflectively in the main model because the three indicators were conceptualized as manifestations of a broader respondent-level perception that structural conditions make international mobility difficult. Under this conceptualization, students perceiving the external mobility environment as more structurally restrictive were expected to report greater difficulty across professional language requirements, relocation costs, and administrative procedures. Nevertheless, these barriers can also occur independently and may alternatively be conceptualized as formative components. Accordingly, the reflective specification is treated as theoretically justified but provisional operationalization that requires comparison with alternative specifications in independent samples.

Migration Readiness (MR) was specified as a formative composite because its four indicators capture distinct and non-interchangeable components of readiness rather than interchangeable manifestations of a single migration intention. MR was therefore estimated as a formative composite within PLS-SEM and was not calculated as an unweighted arithmetic scale. MR1 captures the consideration of working abroad after graduation; MR2 captures the temporal proximity of a potential departure; MR3 reflects perceived professional preparedness to work abroad; and MR4 captures the perception of migration as a realistic solution for career improvement. Professional preparedness (MR3) is not interpreted as evidence of migration intention by itself; rather, it represents the perceived-capability component of readiness. Under the formative specification, a student may feel professionally prepared to work abroad without intending to migrate, while another may consider migration despite not yet feeling sufficiently prepared.

MR1 was coded from 1 = “Not at all” to 5 = “Yes, definitely”, with intermediate categories of 2 = “Probably not”, 3 = “Not sure”, and 4 = “Probably yes”. MR2 was derived from the questionnaire item asking when the respondent would consider leaving if migration were possible. Timing was referenced explicitly to graduation rather than to the date of questionnaire completion. The five response categories were coded as follows: 1 = “I do not intend to leave”; 2 = “More than 5 years after graduation”; 3 = “3–5 years after graduation”; 4 = “1–3 years after graduation”; and 5 = “Immediately after graduation”. Thus, higher values indicate greater temporal proximity to potential migration. MR3 and MR4 were coded directly from 1 = “Strongly disagree” to 5 = “Strongly agree”. Higher analytical values therefore indicate greater Migration Readiness across all four components.

Prior to model estimation, the coding was verified to ensure that higher values reflected a higher level of the corresponding construct.

The instrument was not translated in its entirety from a pre-existing questionnaire; the items were formulated in Romanian and conceptually adapted from the specialized literature. PUSH was grounded in research regarding professional pressures, job satisfaction, retention, and the migration intentions of healthcare personnel. DPO was based on literature concerning professional support, professional recognition, career prospects, and pharmacist retention. PEP drew on studies regarding salaries, working conditions, professional development, and external opportunities. QLP was informed by specialized literature on quality of life and destination attractiveness. PB and SB were based on migration theory, the PPM framework, and research into family, cultural, financial, linguistic, and administrative barriers, while MR was derived from literature regarding intention, aspiration, feasibility, and readiness for mobility. The specific sources used for each construct are presented in Table 2.

The final 26-indicator specification was used to estimate the structural model and for predictive analysis. The constructs are interpreted at an aggregate level: PUSH and DPO describe evaluations of the internal professional context; PEP and QLP describe the attractiveness of external alternatives; PB and SB describe barriers that may limit the translation of interest into mobility; and MR describes general readiness for migration. This architecture allows for the simultaneous examination of the pressures, attractions, and anchors relevant to the retention of the future pharmaceutical workforce.

## 4. Results

### 4.1. Evaluation of the Measurement Model

In the first stage, the measurement model was evaluated through the analysis of outer loadings, internal consistency, convergent and discriminant validity, and collinearity. Since the model includes both reflective and formative constructs, evaluation criteria were applied differentially in accordance with PLS-SEM recommendations [63,74,75].

#### 4.1.1. Evaluation of Reflective Constructs

The results indicate adequate quality for the reflective models; outer loadings range from 0.830 to 0.860 for DPO, 0.846 to 0.880 for PEP, 0.864 to 0.883 for PUSH, 0.856 to 0.892 for QLP, and 0.759 to 0.911 for SB. All loadings were positive and statistically significant at the *p* < 0.001 level. The lowest loading, SB1 = 0.759, remains above the standard threshold of 0.708.

Cronbach’s alpha values range from 0.830 to 0.883, rho_A from 0.848 to 0.907, and rho_C from 0.893 to 0.920, all exceeding the 0.70 threshold. AVE values, ranging from 0.715 to 0.770, exceed the 0.50 threshold; consequently, the reflective constructs demonstrate adequate internal consistency and convergent validity.

#### 4.1.2. Evaluation of Formative Constructs

Cronbach’s alpha, rho_C, and AVE were not interpreted for PB and MR because the indicators represent formative components; the evaluation was based on VIF, weights, loadings, and their statistical significance, as presented in Table 3.

For MR, all four weights are positive and significant. MR4 has the highest relative weight (0.386), followed by MR2 (0.339), MR1 (0.231), and MR3 (0.212), while the outer loadings are all above 0.80 and significant at *p* < 0.001. The result supports the distinct contribution of the four components to the formation of Migration Readiness.

For PB, none of the individual outer weights reaches the 5% significance threshold, although PB4 approaches significance (*p* = 0.051). In contrast, all outer loadings are positive, range from 0.706 to 0.920, and are significant at *p* < 0.001. Because formative outer weights represent relative rather than absolute contributions, their nonsignificance was not considered sufficient grounds for indicator deletion. The indicators were retained because they capture distinct and theoretically relevant forms of personal and social anchoring, exhibit acceptable collinearity, and show significant absolute contributions through their outer loadings. Accordingly, no claim is made that any individual PB component independently dominates the composite, and the PB → MR relationship is interpreted at the composite level. In the absence of redundant global indicators, the formative convergent validity of PB and MR could not be assessed through redundancy analysis and remains a limitation.

#### 4.1.3. Evaluation of Collinearity of Indicators

The VIF values for the outer model range from 1.738 to 2.512; the highest value was observed for PEP2 (2.512), yet all remain below the conservative threshold of 3.3. Consequently, no collinearity issues were identified among the indicators; the VIF values are presented in Table 4.

#### 4.1.4. Assessment of Discriminant Validity

The discriminant validity of the reflective constructs was assessed using the Fornell–Larcker criterion and the HTMT ratio. As PB and MR are formative constructs, they were excluded from this assessment. The square root of the AVE for each construct exceeds its correlations with the other constructs, and HTMT values range from 0.038 to 0.750. The highest value (PEP-QLP = 0.750) falls below the conservative threshold of 0.85, supporting the distinct nature of the two attraction dimensions; the final values are presented in Table 5.

### 4.2. Structural Model Evaluation and Hypothesis Discussion

Prior to interpreting the path coefficients, the collinearity of the inner model was examined. The VIF values range from 1.000 to 1.891 and do not indicate collinearity issues among the predictors of the endogenous constructs. Further, Table 6 present the coefficients of determinations.

**Table 6:** Coefficients of determination.

Endogenous Construct	R^2^	R^2^ Adjusted
DPO	0.150	0.148
PEP	0.259	0.256
QLP	0.471	0.468
MR	0.392	0.384

The model explains 15.0% of the variation in DPO, 25.9% in PEP, 47.1% in QLP, and 39.2% in MR. Its explanatory power is modest for DPO, moderate for PEP, and more substantial for QLP and MR. Given the complexity of migration orientation, these figures suggest that the model captures a significant—though not exhaustive—portion of the variation. Individual factors, preferred destinations, transnational networks, international experience, and family resources could provide further explanatory insights in future models [4,39,47].

The SRMR is 0.037 for the saturated model and 0.038 for the estimated model, both falling below the 0.08 threshold. The NFI for the estimated model is 0.898, very close to 0.90. While these values indicate a reasonably good fit, they are not considered sufficient evidence of model validity; interpretation continues to rely on measurement, structural coefficients, and prediction.

**Figure 2:**
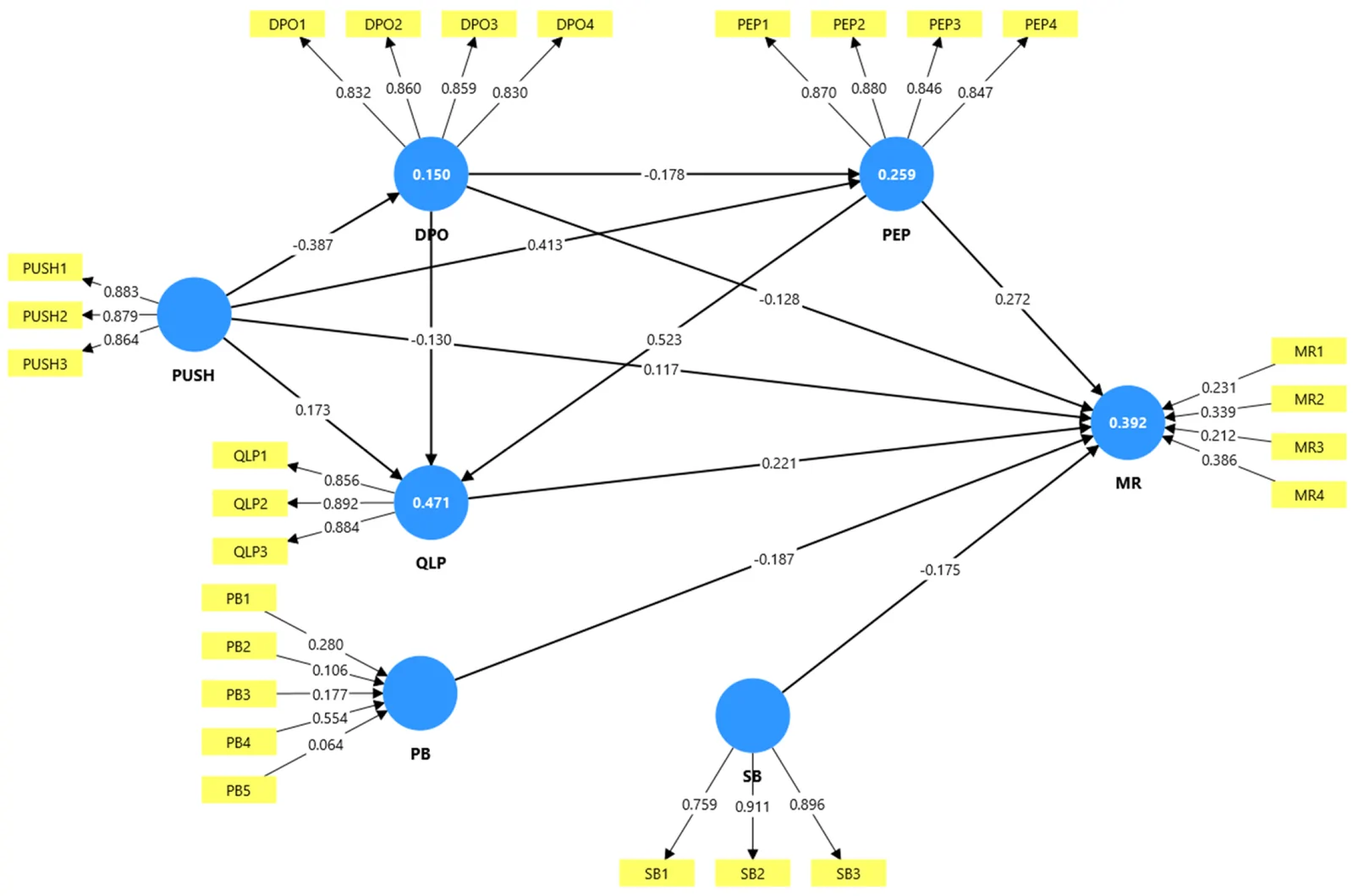
The estimated structural model in SmartPLS.

Table 7 presents the path coefficients and testing of direct hypothesis, confirming that all the hypothesis are validated. Also, the Figure 2 shows the estimated results for our analysis.

**Table 7:** Path coefficients and testing of direct hypotheses.

Hypothesis/Relationship	β	t	*p*	95% CI	f^2^	Results
H1 PUSH → DPO	−0.387	11.245	<0.001	[−0.454; −0.319]	0.176	Supported
H2 PUSH → PEP	0.413	10.817	<0.001	[0.337; 0.486]	0.196	Supported
H3 DPO → PEP	−0.178	4.553	<0.001	[−0.255; −0.102]	0.036	Supported
H4 PUSH → QLP	0.173	4.122	<0.001	[0.090; 0.254]	0.040	Supported
H5 DPO → QLP	−0.130	3.663	<0.001	[−0.201; −0.063]	0.026	Supported
H6 PEP → QLP	0.523	14.107	<0.001	[0.448; 0.594]	0.383	Supported
H7 PUSH → MR	0.117	2.656	0.008	[0.029; 0.202]	0.015	Supported
H8 DPO → MR	−0.128	3.363	0.001	[−0.200; −0.052]	0.021	Supported
H9 PEP → MR	0.272	5.743	<0.001	[0.177; 0.361]	0.065	Supported
H10 QLP → MR	0.221	4.371	<0.001	[0.120; 0.317]	0.043	Supported
H11 PB → MR	−0.187	4.783	<0.001	[−0.265; −0.112]	0.051	Supported
H12 SB → MR	−0.175	5.006	<0.001	[−0.250; −0.114]	0.044	Supported

#### 4.2.1. Internal Professional Pressures and Professional Outlook in Romania

The PUSH→DPO relationship is negative and significant (β = −0.387; t = 11.245; *p* < 0.001), and the f^2^ value of 0.176 indicates a moderate effect, thereby supporting H1. Consequently, the more intensely students perceive a lack of professional prospects, job insecurity, and insufficient social recognition, the more unfavourably they evaluate the future of the pharmacy profession in Romania.

This result aligns with literature linking professional insecurity, remuneration, and career prospects to the external orientation of medical personnel and students. Refs. [4,5] consistently identify these dimensions; ref. [39] demonstrate low satisfaction regarding professional prospects among British students; and ref. [11] report that a negative outlook on a pharmacy career predicts migration intentions. However, the current model introduces a useful distinction: “current pressure” and “professional prospects” are not treated as the same construct, and the coefficient indicates that while they are related, they are not redundant. Literature on the retention of pharmacists and medical personnel shows convergent results regarding the roles of professional prospects, satisfaction, and organizational support [13,14,28,30].

#### 4.2.2. Internal Professional Pressures and External Professional-Economic Attraction

The PUSH → PEP relationship is positive and significant (β = 0.413; t = 10.817; *p* < 0.001); the second hypothesis is supported, and an f^2^ of 0.196 indicates a moderate effect. This result supports the contrast logic of the PPM framework: as professional pressure within the internal environment intensifies, respondents tend to place greater importance on the advantages offered by external opportunities-specifically higher salaries, better working conditions, and professional development prospects. This interpretation aligns with ref. [11], where external opportunities and remuneration were linked to migration intentions, as well as with ref. [39,41], who highlight the role of remuneration and professional development prospects. The result is further supported by reviews conducted by ref. [4,5]. The moderate effect size suggests that PUSH is associated both with MR and with the evaluation of external professional alternatives. This finding is also consistent with pharmaceutical literature regarding occupational stress, job satisfaction, and the intention to leave the profession [8,9,10,51].

#### 4.2.3. Internal Professional Perspective and Professional-Economic Attraction

DPO is negatively associated with PEP (β = −0.178; t = 4.553; *p* < 0.001), supporting the third hypothesis, even though the effect size is indeed small (f^2^ = 0.036).

This direction aligns with the comparative mechanism suggested by recent studies: when internal career opportunities and certainty are perceived favourably, the relative advantage of the external alternative diminishes [3,36,44]. However, the small effect size indicates that a favourable internal outlook does not negate the appeal of external salaries or development opportunities. This is particularly significant for students, who may simultaneously consider a promising internal career path and an attractive external experience. Current literature rarely examines the direct nature of this relationship; thus, the result should be viewed as an extension of existing findings rather than a straightforward application.

#### 4.2.4. Professional Pressures and the Pull of Quality of Life

The PUSH→QLP relationship is positive (β = 0.173; t = 4.122; *p* < 0.001) and significant, supporting the fourth hypothesis; however, the f^2^ value of 0.040 indicates a small effect size. The results show that professional pressures extend beyond salary comparisons: they are also associated with a more favourable assessment of life, culture, and the healthcare system abroad. Refs. [4,40,42] demonstrate that economic, lifestyle, and career-related motives coexist. However, the small effect size suggests that QLP is not merely a buffer against domestic dissatisfaction but also has its own distinct determinants, justifying its separation from the PUSH factor.

#### 4.2.5. Internal Professional Perspective and Attraction Associated with Quality of Life

The relationship between DPO and QLP is negative and significant (β = −0.130; t = 3.663; *p* < 0.001), supporting H5. The effect size is small (f^2^ = 0.026). This finding indicates that a more favourable assessment of domestic professional prospects is associated with a lower perceived attractiveness of quality-of-life opportunities abroad. Although the magnitude of the effect is limited, the result supports the comparative logic of the model: positive expectations regarding one’s professional future in Romania may reduce, but do not eliminate, the perceived advantages associated with living abroad.

#### 4.2.6. Professional-Economic Attraction and Quality of Life Attraction

The relationship between PEP and QLP is the strongest structural relationship in the model (β = 0.523; t = 14.107; *p* < 0.001), and the f^2^ value of 0.383 indicates a large effect size; thus, the sixth hypothesis is supported.

The result suggests an integration of professional and life-related assessments: better wages, working conditions, development opportunities, and access to technology are associated with the anticipation of a more favourable external life experience. Existing literature frequently discusses these benefits together but rarely tests the PEP→QLP sequence; therefore, the model’s contribution lies precisely in formalizing this relationship. Furthermore, the HTMT value of 0.75 indicates that PEP and QLP are closely related yet remain empirically distinct; the high coefficient does not justify combining them into a single construct.

#### 4.2.7. The Direct Effect of Professional Pressures on Migration Readiness

The direct effect of PUSH on MR is positive and significant, indicating that the seventh hypothesis is supported; however, the f^2^ value of 0.015 points to a very small direct effect. This direction aligns with the literature on work pressures, remuneration, and career prospects [4,5,11,36,37]. Yet, this small effect is theoretically more interesting than a large direct effect: it suggests that professional dissatisfaction and migration readiness are related but distinct dimensions. Significant indirect associations involving DPO, PEP, and QLP are consistent with the proposed theoretical architecture, without establishing a temporal decision-making process. The result also aligns with findings from studies on the mobility of pharmacists and medical personnel, which show that internal pressure fosters an orientation toward external alternatives, though the effects are contingent upon opportunities and barriers [3,7,15,60].

#### 4.2.8. Internal Professional Perspective and Migration Readiness

The relationship between DPO and MR is negative and significant (β = −0.128; t = 3.363; *p* = 0.001), supporting H8. The direct effect is small (f^2^ = 0.021).

The result indicates that a favourable internal career outlook acts as a retention factor. This aligns with studies on career intentions and migration that highlight the role of career certainty, development opportunities, and job satisfaction [4,39,43,44,45]. However, the small effect size suggests that a positively perceived career in Romania is not sufficient to eliminate the pull of external opportunities.

#### 4.2.9. Professional-Economic Attraction and Migration Readiness

The relationship between PEP and MR is positive and significant (β = 0.272; t = 5.743; *p* < 0.001), supporting H9. The effect size is small to moderate (f^2^ = 0.065). The result indicates that students who attach greater importance to higher salaries, improved working conditions, professional development opportunities, and access to modern technologies abroad also report higher Migration Readiness. This finding is consistent with evidence showing that professional and economic opportunities represent important components of migration orientation among healthcare professionals and pharmacy-related populations. However, the magnitude of the effect also indicates that professional-economic attraction alone does not fully explain readiness for mobility, which remains dependent on quality-of-life assessments and personal and structural barriers.

#### 4.2.10. Attraction Associated with Quality of Life and Migration Readiness

The QLP → MR relationship is positive and significant (β = 0.221; t = 4.371; *p* < 0.001), supporting H10. The f^2^ value of 0.043 indicates a small effect size.

The result confirms that readiness is not an exclusively economic decision. Factors related to life, well-being, and cultural experience are documented by ref. [4,5,37,39]. The fact that QLP remains significant after the inclusion of PEP demonstrates that the assessment of quality of life provides its own explanatory information. At the same time, the fact that this effect is smaller than that of the PEP → QLP relationship suggests that QLP occupies an intermediate position in the hypothesized statistical model, although its temporal position cannot be established from the present data.

#### 4.2.11. Personal and Social Barriers and Migration Readiness

The relationship PB → MR is negative and significant (β = −0.187; t = 4.783; *p* < 0.001), supporting H11. The value f^2^ = 0.051 indicates a small yet relevant effect.

This result aligns with literature identifying family, social support, alienation, and adaptation as factors that can limit mobility [4,5,42,47,50]. Since PB is formative, the effect must be interpreted at the composite level. Individual weights are not significant at the 5% level, although all loadings are significant; therefore, it cannot be claimed that any specific type of barrier is, in isolation, the primary structural determinant. The relevance of personal and social attachments is supported by literature on migration aspirations and capabilities [36,37,38].

#### 4.2.12. Structural Barriers and Migration Readiness

The SB → MR relationship is negative and significant (β = −0.175; t = 5.006; *p* < 0.001), supporting H12. The f^2^ value of 0.044 indicates a small effect size.

Professional language, costs, and administrative procedures are plausible obstacles to transforming interest into concrete availability. Refs. [42,50] demonstrate the role of language skills and access to information; ref. [49] highlight bureaucratic procedures; and ref. [50,56] document integration and communication barriers faced by migrant staff. The current result indicates that these structural obstacles have an effect independent of PB, supporting the distinction between the two categories of “mooring.” Studies on the integration of migrant medical staff show that language, qualification recognition, costs, and institutional adaptation can act as independent constraints on mobility [12,56].

#### 4.2.13. Indirect Effects and Mediations

Hypothesis 13 is supported, as can be seen in Table 8; the PEP → QLP → MR effect is positive and significant (β = 0.116; *p* < 0.001). The direct effect of PEP on MR also remains positive and significant (β = 0.272), and the total effect of PEP on MR is 0.388. The estimated indirect association through QLP represents approximately 29.9% of the total association; this proportion is descriptive and does not establish causal mediation. This configuration is consistent with complementary partial mediation.

**Table 8:** Specific Indirect Effects and Testing of Mediation Hypotheses.

Hypothesis/Indirect Effect	β	t	*p*	95% CI	Results
H13 PEP → QLP → MR	0.116	4.174	<0.001	[0.062; 0.171]	Supported
H14a PUSH → PEP → MR	0.112	4.863	<0.001	[0.069; 0.159]	Supported
H14b PUSH → QLP → MR	0.038	3.002	0.003	[0.016; 0.065]	Supported
H14c PUSH → PEP → QLP → MR	0.048	3.832	<0.001	[0.025; 0.074]	Supported
H15a DPO → PEP → MR	−0.048	3.482	<0.001	[−0.078; −0.024]	Supported
H15b DPO → QLP → MR	−0.029	2.764	0.006	[−0.052; −0.011]	Supported
H15c DPO → PEP → QLP → MR	−0.021	3.020	0.003	[−0.036; −0.009]	Supported
H16a PUSH → DPO → MR	0.050	3.209	0.001	[0.020; 0.080]	Supported
H16b PUSH → DPO → PEP → MR	0.019	3.287	0.001	[0.009; 0.031]	Supported
H16c PUSH → DPO → QLP → MR	0.011	2.615	0.009	[0.004; 0.021]	Supported
H16d PUSH → DPO → PEP → QLP → MR	0.008	2.846	0.004	[0.003; 0.014]	Supported

From a theoretical perspective, this demonstrates that professional-economic advantages are linked to readiness, both directly and through the anticipation of a better quality of life. While the literature frequently acknowledges the existence of these benefits, it rarely explicitly tests this sequence; thus, the result represents an advancement of the PPM mechanism rather than a mere replication [4,5,28,37,39,40].

For PUSH factors, the total indirect effect on MR is 0.286, while the direct effect is 0.117 and the total effect is 0.402. Approximately 71% of the total effect is indirect. The pathways PUSH → PEP → MR, PUSH → QLP → MR, and PUSH → PEP → QLP → MR are significant, as are the routes involving DPO. This result reinforces the interpretation that professional pressures are associated with readiness both directly and indirectly through domestic professional outlook and the perceived attractiveness of external opportunities. This conclusion aligns with the PPM literature and research distinguishing dissatisfaction from the attractiveness of external options [4,5] as well as with the study by ref. [46], which shows that the relationship between professional conditions and emigration can be mediated by psychosocial mechanisms. For DPO, the indirect effect on readiness is −0.098, the direct effect is −0.128, and the total effect is −0.226. The estimated indirect associations involving PEP and QLP account for approximately 43.3% of the total association. All three proposed pathways—via PEP, via QLP, and serially via PEP and QLP—are significant. This configuration indicates that a favourable internal perspective is associated with lower readiness, both directly and through the reduction of the comparative attractiveness of external opportunities. However, given the cross-sectional design, the mediation proportions are descriptive, and the sequence should not be interpreted as a demonstrated temporal mechanism.

### 4.3. Sensitivity Analysis of the Migration Readiness Specification

To assess whether the structural findings depended on the inclusion of perceived professional preparedness (MR3), the complete model was re-estimated after excluding MR3 from the formative MR composite. The explanatory power for MR remained highly similar (R^2^ = 0.383 compared with 0.392 in the main model). The direction and statistical significance of all six direct associations with MR were preserved: PUSH (β = 0.115, *p* = 0.009), DPO (β = −0.134, *p* < 0.001), PEP (β = 0.259, *p* < 0.001), QLP (β = 0.223, *p* < 0.001), PB (β = −0.192, *p* < 0.001), and SB (β = −0.169, *p* < 0.001). The remaining formative MR indicators also retained significant outer weights and acceptable VIF values. These findings indicate that the substantive structural conclusions are robust to the exclusion of the professional-preparedness component of MR. Detailed results of the sensitivity analysis, including the comparation of structural associations, measurement properties of the reduced MR composite, and the specific indirect effects, are provided in Supplementary Tables S1–S3.

### 4.4. Predictive Performance Evaluation

In addition to explanatory power, internal cross-validated predictive performance was evaluated using PLSpredict with 10 folds and 10 repetitions. The Q^2^predict values are positive for all indicators of the four endogenous constructs, indicating that the PLSpredict predictions outperform the benchmark based on indicator means, as can be seen in Table 9. At the construct level, Q^2^predict is 0.146 for DPO, 0.228 for PEP, 0.222 for QLP, and 0.184 for MR.

For the DPO, PEP, and QLP indicators, the RMSE is lower in PLS-SEM than in the linear model for all variables. For MR, the comparison is mixed: PLS-SEM shows a marginally lower RMSE for MR2 and MR4, but a marginally higher one for MR1 and MR3; MAE values are slightly higher in PLS-SEM for all four MR indicators. Consequently, the predictive relevance of MR is positive, but the advantage over the linear model is not uniform across the indicators.

CVPAT confirms that PLS-SEM exhibits significantly lower predictive loss than the indicator-mean-based benchmark for DPO, PEP, QLP, MR, and the overall model can be seen in Table 10. Compared to the linear model, the advantage is significant for DPO (*p* = 0.024), PEP (*p* = 0.005), QLP (*p* < 0.001), and the overall model (*p* < 0.001). However, for MR, the difference is not significant (ΔLoss = 0.003; *p* = 0.839).

The interpretation must, therefore, be nuanced. The model demonstrates positive predictive relevance for Migration Readiness and clearly outperforms a naive benchmark, yet it does not offer a significant predictive advantage over linear regression for MR. In contrast, the model’s overall performance is significantly better than that of the linear benchmark due to the prediction of DPO, PEP, and QLP. This distinction is important because a moderate R^2^ value or a positive Q^2^predict does not automatically imply superiority over an alternative predictive model [67,68,69].

### 4.5. Summary of Results and Relevance for the Healthcare Workforce

From a healthcare-workforce perspective, the findings indicate that Migration Readiness is associated with a combination of internal professional pressures, external attractiveness, and mobility constraints. PUSH is negatively associated with DPO and positively associated with PEP and QLP, while its direct association with MR is comparatively small. The significant specific indirect associations are consistent with the proposed PPM architecture, in which professional pressures are related to MR both directly and through domestic career outlook and the perceived attractiveness of external alternatives. However, because the data are cross-sectional, these associations do not establish temporal ordering or causality.

The relationship between PEP and QLP is the strongest in the model and carries significant implications for retention policies. Salary benefits, working conditions, professional development, and access to technology are not perceived in isolation from the overall quality of life. Consequently, the competitiveness of the pharmaceutical workforce cannot be built through a single intervention, such as adjusting remuneration. Retention is more likely to depend on a coherent package that combines career prospects, high-value-added professional roles, working conditions, predictability, work-life balance, and professional recognition. More favourable domestic professional outlook is associated with lower perceived external attractiveness and lower Migration Readiness.

Personal barriers (PB) and structural barriers (SB) complement this explanation by introducing the dimension of feasibility. The distinct negative effects on MR confirm that migration readiness is not equivalent to the attractiveness of working abroad. From a human resource management perspective, this distinction is crucial; readiness indicators can identify latent pressures on retention but should not be interpreted as individual probabilities of departure. The model explains 39.2% of the variation in MR and exhibits a low SRMR, with positive predictive relevance; however, CVPAT does not confirm its superiority over the linear model for MR. These findings justify further investigation of MR as a candidate aggregate perceptual indicator of retention vulnerability. Its operational use for monitoring or prioritizing interventions requires longitudinal and external validation against actual employment and migration outcomes.

## 5. Conclusions

The study aimed to explain pharmacy students’ willingness to migrate and to assess its relevance for the retention and planning of the future pharmaceutical workforce. An extended Push-Pull-Mooring model was employed, estimated via PLS-SEM using 520 valid questionnaires; the measurement model, structural relationships, and indirect effects were evaluated using bootstrapping with 10,000 subsamples. The explanatory analysis was complemented by PLSpredict and CVPAT, allowing the model’s utility to be examined not only through the statistical significance of relationships but also through its internal cross-validated predictive performance.

Higher perceived internal professional pressures are associated with less favourable domestic professional outlook and greater perceived attractiveness of external alternatives. The PEP → QLP relationship is the strongest structural association in the model (β = 0.523), and both PEP and QLP are positively associated with MR. DPO, PB, and SB are negatively associated with MR. The model explains 39.2% of the variance in MR and shows a low estimated SRMR of 0.038. Significant indirect associations involving domestic professional outlook and external attractiveness are consistent with the proposed PPM architecture, but do not establish temporal ordering or causality. MR may therefore be considered a candidate aggregate perceptual indicator of future workforce-retention vulnerability. Its operational value requires longitudinal and external validation against actual post-graduation employment and migration outcomes.

### 5.1. Theoretical Implications

The first theoretical implication is the extension of the PPM framework from individual mobility to the analysis of retention vulnerability within the pre-professional stage of the healthcare workforce. Most PPM applications address active populations whose migration behaviors are already close to the decision-making stage. The present results demonstrate that “push,” “pull,” and “mooring” mechanisms can also be conceptualized within the educational pipeline, prior to graduates fully entering the healthcare system. This shift is significant because healthcare workforce planning depends not only on the current stock of professionals but also on the likelihood that the flow of graduates will be converted into human resources available to the national system.

The second contribution is the distinction between “Pull” factors categorized as PEP (Professional-Economic Pull) and QLP (Quality-of-Life Pull). The results show that professional-economic advantages and quality-of-life benefits are empirically distinct yet strongly interconnected. The relationship between PEP and QLP suggests that the assessment of salaries, working conditions, and professional development is associated with more favourable perceptions of life abroad. This structure offers a more nuanced explanation than models in which all destination-related benefits are aggregated into a single “Pull” construct.

The third contribution is the introduction of DPO (Domestic Professional Outlook) as a distinct retention mechanism. The results indicate that a favorable domestic professional outlook is associated with lower Migration Readiness (MR) both directly and through negative indirect associations involving PEP and QLP. Theoretically, this suggests that retention is not merely the absence of “push” factors but can result from a positive and credible assessment of one’s professional future in the home country. Furthermore, the separation of personal and social barriers (PB) from structural barriers (SB) supports the idea that these operate as distinct anchoring mechanisms; this aligns with the aspirations-capabilities perspective, implying that the desire for mobility and the capacity to achieve it are not equivalent.

Moreover, the indirect and serial effects support interpreting the PPM as a process rather than merely a set of parallel predictors. From the perspective of retention theory, the observed associations are consistent with a framework in which professional pressures are related to MR both directly and indirectly through domestic professional outlook and external attractiveness; the present design does not establish these relationships as causal mechanisms. This framework bridges migration literature with workforce retention literature, providing a basis for conceptualizing MR as an intermediary indicator linking career perceptions to potential human capital loss. However, the cross-sectional design necessitates interpreting these pathways as structural associations consistent with the proposed mechanism, rather than as proven causal relationships.

### 5.2. Practical Implications

For universities and pharmacy faculties, the results suggest that the retention of future professionals can be supported prior to graduation through a clearer transition between education and healthcare practice. Interventions should not discourage international mobility but rather enhance the visibility and appeal of a competitive pharmaceutical career in Romania. Providing a realistic overview of career paths—spanning community and hospital pharmacy, industry, research, administration, and academia—alongside connecting students with mentors and employers and offering exposure to clinical roles, can reduce uncertainty regarding professional futures and strengthen professional commitment.

For organizations providing pharmaceutical services, the PEP and QLP results indicate the need for a retention package that extends beyond remuneration. Onboarding programs, mentoring, continuous professional development, access to technology and clinical services, transparent promotion criteria, predictable scheduling, and work-life balance can collectively influence the attractiveness of internal career paths. Such an approach is also relevant to service quality, as the continuity and skill development of the pharmacy workforce determine an organization’s capacity to expand patient-centered services and support the safe use of medicines.

At the managerial level, MR-based measures could be explored in aggregate form for organizational and educational monitoring, provided that their longitudinal validity is established in future research. Faculties, hospital pharmacies, community pharmacy organizations, and other stakeholders could monitor career perspectives, pull factors, and barriers over time and assess changes following retention-oriented interventions. The present findings do not support individual-level classification or prediction of persons at risk of leaving; their potential practical value lies in identifying areas of aggregate retention pressure that may warrant further investigation.

### 5.3. Implications for Government Policies

The results are directly relevant to health workforce planning policies. Monitoring pharmaceutical human resources should not be limited to the number of registered pharmacists, vacancies, or geographical distribution. Perceptual measures such as DPO, the attractiveness of external opportunities, and MR may provide preliminary aggregate signals of retention pressure among students preparing to enter the profession. Their future integration with longitudinal and administrative workforce data could support earlier identification of potential vulnerabilities; however, the present cross-sectional findings do not establish MR as a validated early-warning tool.

Public policies should prioritize structural factors that the system can influence. More favourable domestic career prospects are associated with lower MR, both directly and through negative indirect associations involving the attractiveness of external opportunities. Consequently, relevant measures include clearer career paths, the expansion of pharmacists’ clinical roles, integration into multidisciplinary teams, recognition and funding for clinically valuable pharmacy services, access to specialization and continuing education, and the facilitation of participation in research and innovation. A strategy focused exclusively on salary would be insufficient, as PEP and QLP are interdependent.

The Ministry of Health, the Ministry of Education, professional organizations, and universities could develop a joint mechanism to monitor the pharmaceutical workforce pipeline—from student to graduate and, subsequently, to active professional. Data on medical migration could be correlated, in aggregated form, with the number of graduates, professional association membership, field of practice, geographic distribution, and outward mobility. Such an infrastructure would allow for an assessment of the gap between intention and actual behaviour, providing a more solid basis for planning jobs and training positions, designing incentives for underserved areas, and formulating retention policies.

At the same time, retention policies must respect freedom of movement and the benefits of professional mobility. The goal is not to restrict migration, but to reduce migration driven by a lack of credible domestic prospects and to create conditions that make staying, returning, or circular mobility viable options. Institutional partnerships, as well as programs for return and the recognition of skills acquired abroad, can transform mobility from a one-sided loss into a mechanism for transferring skills to the national health system.

### 5.4. Research Limitations and Future Directions

The results must be interpreted in light of several limitations. First, the cross-sectional design precludes the establishment of a definitive temporal sequence or the formulation of causal conclusions. The estimated relationships and mediations indicate associations consistent with the theoretical model but do not demonstrate that a change in a predictor subsequently causes a change in migration readiness (MR). Longitudinal studies involving repeated measurements—taken during the course of study and in the initial years following graduation—could test whether the identified relationships actually predict migration decisions.

Second, online recruitment and the non-probabilistic nature of the sample limit the generalizability of the findings to the entire population of pharmacy students in Romania. Future research should employ stratified sampling based on universities, years of study, and regions, and compare university centres with different profiles. Multi-group analyses could examine differences based on gender, year of study, international experience, urban/rural background, or the intention to pursue specific sectors of the pharmacy profession.

Third, MR is operationalized as a formative composite combining stated intention, temporal proximity, perceived professional preparedness, and migration realism. Although this specification captures the multidimensional nature of readiness, it requires further validation using independent samples. Future research could compare this specification with alternative models that separate behavioural intention, migration self-efficacy, and perceived feasibility, or that utilize a redundant global indicator for the convergent validation of the composite. Fourthly, the model does not include all variables relevant to the migration decision. Future research could incorporate professional identity, satisfaction with the study program, academic burnout, family income level, the presence of relatives or social networks abroad, Erasmus experience, actual language proficiency, preferred destination country, and the perceived recognition of qualifications. Analysing how these characteristics moderate the relationships could identify subgroups for which the push, pull, and mooring mechanisms operate differently.

Fifthly, the model’s predictive performance requires external validation and linkage to actual workforce outcomes. Although the Q^2^predict values are positive and the model outperforms the naive benchmark, CVPAT does not demonstrate significant superiority over the linear model for MR. The most important future direction involves the longitudinal tracking of student cohorts’ post-graduation and—where ethical and legal frameworks permit—linking aggregated responses with administrative data on professional integration, employment sector, geographical distribution, and actual migration. Such validation would enable the estimation of workforce retention value. Replication in other countries could test whether the PEP → QLP → MR architecture reflects a general mechanism of future pharmacist mobility or is specific to the Romanian context.

Although the discriminant-validity criteria supported the empirical separation of PUSH and DPO, their conceptual proximity warrants replication using alternative item formulations and longitudinal designs. The reflective specification of SB should also be reassessed against a formative alternative in independent samples. University/Faculty affiliation was self-reported and was not independently verified against institutional administrative records. Although the distribution of respondents by university centre is reported in Table 1, recruitment was not based on institutional quotas or probability sampling. The sample therefore does not support institutionally or nationally representative estimates. Finally, although the sensitivity analysis showed that the structural conclusions remained stable after excluding MR3, the formative operationalization of MR requires further longitudinal and external validation against actual post-graduation employment and migration outcomes.

Overall, the findings support MR as a multidimensional formative construct capturing migration consideration, temporal proximity, perceived professional capability, and perceived career feasibility. At the aggregate level, MR may represent a candidate perceptual indicator of future pharmaceutical workforce-retention vulnerability. However, its operational use in workforce planning requires longitudinal and external validation against actual transition-to-practice, employment, and migration outcomes. Accordingly, the present findings should be interpreted as evidence of cross-sectional associations relevant to workforce-retention research rather than as individual-level predictions of future migration.

## Figures and Tables

**Table 1:** Socio-demographic characteristics of the sample.

Variable	Category	N	%
Gender	Female	353	67.9%
	Male	167	32.1%
Background	Rural	173	33.3%
	Urban	347	66.7%
Marital status	Unmarried	470	90.4%
	Married	50	9.6%
Relatives/close friends abroad	Yes	417	80.2%
	No	103	19.8%
Academic/professional experience abroad	Yes	135	26.0%
	No	385	74.0%
Year of study	1st Year	67	12.9%
	2nd Year	91	17.5%
	3rd Year	120	23.1%
	4th Year	128	24.6%
	5th Year	114	21.9%
Funding type	Fee-paying	118	22.7%
	Tuition-free	402	77.3%
University centre	Iași	218	41.92%
	Bucharest	179	34.42%
	Craiova	32	6.15%
	Oradea	30	5.77%
	Târgu Mureș	27	5.19%
	Cluj-Napoca	18	3.46%
	Galați	16	3.08%

**Table 2:** Model constructs, indicators and psychometric properties.

Indicator	Meaning of the Indicator	Cronbach’s Alpha	Rho_A	Rho_C	AVE
PUSH—Professional push factors. Reflective construct (Mode A), developed and adapted by the authors based on ref. [3,4,5,11,40,46].					
PUSH1	Lack of professional career prospects in Romania	0.848	0.848	0.908	0.767
PUSH2	Job insecurity in Romania				
PUSH3	Lack of social recognition of the pharmacy profession				
DPO—Domestic Professional Outlook. Reflective construct (Mode A), adapted by the authors based on ref. [13,14,29,30,32]					
DPO1	Satisfaction with the professional prospects of pharmacists in Romania	0.867	0.867	0.909	0.715
DPO2	Perception that the pharmacy profession is appreciated in Romania				
DPO3	Perception that the future of the pharmacy profession is promising in Romania				
DPO4	Perception that the Romanian healthcare system supports young pharmacists				
PEP—Professional-Economic Pull. Reflective construct (Mode A), adapted by the authors based on ref. [3,4,5,11,12,42].					
PEP1	Possibility of obtaining a higher salary abroad	0.883	0.884	0.920	0.741
PEP2	Possibility of benefiting from better working conditions				
PEP3	Greater opportunities for professional development				
PEP4	Access to modern technology and research				
QLP—Quality-of-Life Pull. Reflective construct (Mode A), developed and adapted by the authors based on ref. [3,4,37,38,42].					
QLP1	Possibility of experiencing another culture	0.851	0.852	0.910	0.770
QLP2	Possibility of achieving a higher quality of life				
QLP3	Perception of a higher-quality healthcare system				
SB—Structural Barriers. Reflective construct (Mode A), developed and adapted by the authors based on ref. [4,5,36,42,57].					
SB1	Lack of professional-level foreign language proficiency	0.830	0.907	0.893	0.736
SB2	High costs associated with relocation				
SB3	Complexity of administrative procedures				
PB—Personal and Social Barriers. Formative construct (Mode B), developed and adapted by the authors based on ref. [4,5,23,37,42].					
PB1	Fear of adapting to a new culture	–	–	–	–
PB2	Desire to remain close to family and friends				
PB3	Uncertainty regarding career stability abroad				
PB4	Current geopolitical context				
PB5	Current economic context				
MR—Migration Readiness. Formative construct (Mode B), adapted by the authors based on ref. [4,5,11,36,37,38,42].					
MR1	Considering working abroad after graduation	–	–	–	–
MR2	Temporal proximity of a possible departure				
MR3	Perceived professional readiness to work abroad				
MR4	Migration perceived as a realistic solution for career improvement				

**Table 3:** Evaluation of formative constructs.

Indicator	VIF	Weight	t	*p*	Loading	*p* Loading
MR1	2.153	0.231	2.474	0.013	0.828	<0.001
MR2	2.206	0.339	4.009	<0.001	0.872	<0.001
MR3	2.028	0.212	2.428	0.015	0.807	<0.001
MR4	2.182	0.386	3.858	<0.001	0.887	<0.001
PB1	2.021	0.280	0.877	0.380	0.814	<0.001
PB2	2.387	0.106	0.326	0.744	0.784	<0.001
PB3	2.105	0.177	0.528	0.598	0.762	<0.001
PB4	1.979	0.554	1.955	0.051	0.920	<0.001
PB5	1.990	0.064	0.204	0.838	0.706	<0.001

**Table 4:** VIF intervals for the external model.

Indicators	VIF Range
DPO1–DPO4	1.907–2.239
PEP1–PEP4	2.109–2.512
PUSH1–PUSH3	1.919–2.199
QLP1–QLP3	1.908–2.246
SB1–SB3	1.738–2.118
PB1–PB5	1.979–2.387
MR1–MR4	2.028–2.206

**Table 5:** Discriminant Validity Assessment: Fornell–Larcker Criterion and HTMT.

Fornell–Larcker					Construct	HTMT				
DPO	PEP	PUSH	QLP	SB		DPO	PEP	PUSH	QLP	SB
0.845					DPO					
−0.337	0.861				PEP	0.386				
−0.387	0.482	0.876			PUSH	0.450	0.557			
−0.373	0.650	0.475	0.878		QLP	0.434	0.750	0.559		
−0.006	0.062	0.119	0.033	0.858	SB	0.038	0.084	0.138	0.049	

Note: The values on the diagonal of the Fornell–Larcker matrix represent the square roots of the AVE values. PB and MR are not included in this assessment because they were specified formatively (Mode B).

**Table 9:** PLSpredict results at the level of endogenous indicators.

Indicator	Q^2^predict	RMSE PLS	RMSE LM	MAE PLS	MAE LM
DPO1	0.085	1.145	1.164	0.957	0.976
DPO2	0.132	1.115	1.127	0.932	0.940
DPO3	0.094	1.139	1.151	0.950	0.954
DPO4	0.104	1.133	1.145	0.932	0.934
PEP1	0.161	1.027	1.038	0.823	0.828
PEP2	0.179	1.016	1.029	0.823	0.829
PEP3	0.160	1.027	1.041	0.831	0.841
PEP4	0.174	1.019	1.031	0.827	0.838
QLP1	0.152	1.033	1.055	0.831	0.847
QLP2	0.160	1.027	1.041	0.815	0.827
QLP3	0.201	1.002	1.021	0.812	0.820
MR1	0.115	1.135	1.129	0.937	0.923
MR2	0.141	1.118	1.120	0.915	0.910
MR3	0.119	1.133	1.131	0.929	0.928
MR4	0.159	1.107	1.109	0.923	0.914

**Table 10:** CVPAT results at the level of endogenous constructs.

Construct	Benchmark	PLS Loss	Benchmark Loss	Δ Loss	t	*p*
DPO	Indicator average	1.284	1.433	−0.149	4.866	<0.001
PEP	Indicator average	1.045	1.257	−0.212	5.511	<0.001
QLP	Indicator average	1.043	1.257	−0.215	5.481	<0.001
MR	Indicator average	1.262	1.457	−0.194	5.146	<0.001
General model	Indicator average	1.166	1.357	−0.191	7.216	<0.001
DPO	Linear model	1.284	1.315	−0.031	2.267	0.024
PEP	Linear model	1.045	1.071	−0.026	2.799	0.005
QLP	Linear model	1.043	1.080	−0.037	5.641	<0.001
MR	Linear model	1.262	1.260	0.003	0.203	0.839
Overall model	Linear model	1.166	1.188	−0.022	3.362	<0.001

## Data Availability

The data presented in this study are available on request from the corresponding authors. The data are not publicly available due to privacy and ethical restrictions.

## Supplementary Materials

The following supporting information can be downloaded at: https://www.mdpi.com/article/10.3390/healthcare14213745/s1, Supplementary Table S1: Sensitivity analysis excluding MR3 from the Migration Readiness composite; Supplementary Table S2: Measurement properties of the Migration Readiness composite in the sensitivity model; Supplementary Table S3: Comparison of specific indirect effects between the main and sensitivity models; Supplementary File S1: Full English-language questionnaire; Supplementary File S2: The data base of the results of questionnaire.

## Abbreviations

The following abbreviations are used in this manuscript:

PPM	Push–Pull–Mooring
PLS-SEM	Partial Least Squares Structural Equation Modeling
PUSH	Professional Push Factors
DPO	Domestic Professional Outlook
PEP	Professional-Economic Pull
QLP	Quality-of-Life Pull
PB	Personal and Social Barriers
SB	Structural Barriers
MR	Migration Readiness
VIF	Variance Inflation Factor
AVE	Average Variance Extracted
HTMT	Heterotrait–Monotrait Ratio
SRMR	Standardized Root Mean Square Residual
NFI	Normed Fit Index
RMSE	Root Mean Square Error
MAE	Mean Absolute Error
CVPAT	Cross-Validated Predictive Ability Test
R^2^	Coefficient of Determination
Q^2^predict	Predictive Relevance Statistic
WHO	World Health Organization
