# Supplementary material for: Drivers of Migration Readiness Among Romanian Pharmacy Students: An Extended Push–Pull–Mooring Model
Source: Healthcare (Basel). 2026 Nov 3;14(21):3745. doi: 10.3390/healthcare14213745 (PMC13762563; doi:10.3390/healthcare14213745)
Supplement: Supplementary file 1 [file healthcare-14-03745-s001.zip › File S1_Questionnaire_Pharmacy_Students_English_Aligned_with_Manuscript.pdf]

RESEARCH

This research is conducted by faculty members of the "Grigore T. Popa" University of Medicine and Pharmacy of Iași, Faculty of Pharmacy, under the coordination of Professor Cătălina Stan. We aim to identify the intentions, motivations, and barriers related to professional migration (moving abroad to work as a pharmacist) among pharmacy students in Romania. Participation is voluntary, and responses are confidential and anonymous. Completing the questionnaire takes approximately 8–12 minutes and is not expected to cause psychological or social discomfort. By completing the questionnaire, you confirm that you have understood the purpose of the study and agree to participate. All information you provide will be treated as confidential and used solely for research purposes. The study results may be presented at scientific events or published in academic works without disclosing personal data that could allow any participant to be identified.

Pursuant to the Universal Declaration of Human Rights, Government Ordinance No. 57/2002 on scientific research and technological development, approved by Law No. 324/2003, as well as the Code of Ethics of the pharmacist profession, as a participant in this study you have the right to refuse participation and the right to request explanations and clarifications regarding the aims, procedures, or results of the study in which you participate.

** Indicates a required question*

If you agree to participate in the research, please indicate your consent below. ☐

I agree

QUESTIONNAIRE – PROFESSIONAL MIGRATION AND MIGRATION READINESS
AMONG PHARMACY STUDENTS IN ROMANIA

English-language version aligned with the final construct specification used in the manuscript

SECTION A. SOCIO-DEMOGRAPHIC AND ACADEMIC PROFILE

1. Gender:

☐ Female

☐ Male

2. Age: ____ years

3. University / Faculty: ____

4. Place of residence:

☐ Urban

☐ Rural

5. Marital status:

☐ Unmarried

☐ Married

6. Do you have relatives or close friends living abroad?

☐ Yes

☐ No

7. Have you had academic or professional experience abroad (internships, Erasmus, etc.)?

☐ Yes

☐ No

8. Year of study: ___________

9. Funding status:

☐ Fee-paying

☐ Tuition-free

10. Have you considered working abroad as a pharmacist after completing your studies?

☐ Yes, definitely

☐ Probably yes

☐ Not sure

☐ Probably not

☐ Not at all

11. If it were possible, when would you consider leaving?

☐ Immediately after graduation

☐ 1–3 years after graduation

☐ 3–5 years after graduation

☐ More than 5 years after graduation

☐ I do not intend to leave

12. In which country would you most like to work as a pharmacist?

☐ Germany

☐ France
☐ United Kingdom
☐ United States
☐ Other country (please specify): ____________________

SECTION B. FACTORS ASSOCIATED WITH WORKING ABROAD

Instruction: Please rate how important each of the following factors would be in your decision to work abroad as a pharmacist, using a scale from 1 to 5 (1 = not at all important, 2 = slightly important, 3 = important, 4 = quite important, 5 = very important).

No.	Factor	1	2	3	4	5
13.	Possibility of obtaining a higher salary abroad	☐	☐	☐	☐	☐
14.	Possibility of benefiting from better working conditions (equipment, working schedule)	☐	☐	☐	☐	☐
15.	Greater opportunities for professional development	☐	☐	☐	☐	☐
16.	Access to modern technology and research	☐	☐	☐	☐	☐
17.	Possibility of experiencing another culture	☐	☐	☐	☐	☐
18.	Possibility of achieving a higher quality of life	☐	☐	☐	☐	☐
19.	A higher-quality healthcare system	☐	☐	☐	☐	☐
20.	Opportunity to start a family in a different environment	☐	☐	☐	☐	☐
21.	Lack of professional career prospects in Romania	☐	☐	☐	☐	☐
22.	Job insecurity in Romania	☐	☐	☐	☐	☐
23.	Lack of social recognition of the pharmacy profession in Romania	☐	☐	☐	☐	☐

SECTION C. BARRIERS TO PROFESSIONAL MIGRATION

Instruction: Please rate the extent to which each of the following perceived difficulties could prevent you from working abroad as a pharmacist, using a scale from 1 to 5 (1 = not a difficulty, 2 = a minor difficulty, 3 = a difficulty, 4 = a major difficulty, 5 = a very major difficulty).

No.	Potential barrier	1	2	3	4	5
24.	Lack of professional-level foreign language proficiency	☐	☐	☐	☐	☐
25.	High costs associated with relocation	☐	☐	☐	☐	☐
26.	Complexity of administrative procedures (e.g., recognition of the degree/professional qualification)	☐	☐	☐	☐	☐

27.	Fear of adapting to a new culture	☐	☐	☐	☐	☐
28.	Desire to remain close to family and friends	☐	☐	☐	☐	☐
29.	Uncertainty regarding career stability abroad	☐	☐	☐	☐	☐
**No.**	**Potential barrier**	**1**	**2**	**3**	**4**	**5**
30.	Current geopolitical context	☐	☐	☐	☐	☐
31.	Current economic context	☐	☐	☐	☐	☐
32.	My partner has a job/profession for which there is no equivalent abroad	☐	☐	☐	☐	☐

SECTION D. PROFESSIONAL OUTLOOK, RETENTION, AND MIGRATION READINESS

33. To what extent are you satisfied with the professional prospects of pharmacists in Romania?

☐ To a very great extent
☐ To a great extent
☐ Neutral
☐ To a small extent
☐ To a very small extent

34. To what extent do you consider that the recognition of pharmaceutical services contributes to increasing the prestige of the pharmacy profession in Romania?

☐ To a very great extent
☐ To a great extent
☐ Neutral
☐ To a small extent
☐ To a very small extent

35. From the options below, select the main reason that would encourage you to remain in Romania:

☐ More competitive salaries
☐ Improved working conditions
☐ Continuing education and specialization opportunities
☐ Mentoring and professional integration programs
☐ Professional recognition and social respect
☐ Financial support at the beginning of the career
☐ Other reason: ________________________

Instruction: Please indicate your level of agreement with each statement on a scale from 1 to 5 (1 = strongly disagree, 2 = disagree, 3 = neutral, 4 = agree, 5 = strongly agree).

No.	Statement	1	2	3	4	5
36.	The pharmacy profession is appreciated in Romania	☐	☐	☐	☐	☐
37.	The future of the pharmacy profession in Romania is promising	☐	☐	☐	☐	☐

38.	I feel professionally prepared to work abroad	☐	☐	☐	☐	☐
39.	The Romanian healthcare system provides sufficient support to young pharmacists	☐	☐	☐	☐	☐
40.	If salaries were competitive, I would prefer to remain in Romania	☐	☐	☐	☐	☐

No.	Statement	1	2	3	4	5
41.	I consider migration a realistic solution for improving my career	☐	☐	☐	☐	☐

SECTION E. EXPLORATORY PERSONALITY ITEMS

Please indicate how characteristic each statement is of you at the time you complete the questionnaire, using a scale from 1 to 5 (1 = not at all characteristic, 2 = slightly characteristic, 3 = neutral, 4 = characteristic, 5 = very characteristic).

No.	Item	1	2	3	4	5
42.	I am the life of the party	☐	☐	☐	☐	☐
43.	I do not talk a lot	☐	☐	☐	☐	☐
44.	I talk to many different people at parties	☐	☐	☐	☐	☐
45.	I keep in the background	☐	☐	☐	☐	☐
46.	I feel others' emotions	☐	☐	☐	☐	☐
47.	I am not interested in other people's problems	☐	☐	☐	☐	☐
48.	I have a soft heart	☐	☐	☐	☐	☐
49.	I insult people	☐	☐	☐	☐	☐
50.	I am always prepared	☐	☐	☐	☐	☐
51.	I leave my belongings around	☐	☐	☐	☐	☐
52.	I pay attention to details	☐	☐	☐	☐	☐
53.	I make a mess of things	☐	☐	☐	☐	☐
54.	I get stressed out easily	☐	☐	☐	☐	☐
55.	I am relaxed most of the time	☐	☐	☐	☐	☐
56.	I worry about things	☐	☐	☐	☐	☐
57.	I seldom feel blue	☐	☐	☐	☐	☐
58.	I have a rich vocabulary	☐	☐	☐	☐	☐
59.	I have difficulty understanding abstract ideas	☐	☐	☐	☐	☐
60.	I have a vivid imagination	☐	☐	☐	☐	☐
61.	I am not interested in abstract ideas	☐	☐	☐	☐	☐
