# Supplementary material for: Drivers of Migration Readiness Among Romanian Pharmacy Students: An Extended Push–Pull–Mooring Model
Source: Healthcare (Basel). 2026 Nov 3;14(21):3745. doi: 10.3390/healthcare14213745 (PMC13762563; doi:10.3390/healthcare14213745)
Supplement: Supplementary file 1 [file healthcare-14-03745-s001.zip › Supplementary Tables.pdf]

Supplementary Table S1. Sensitivity Analysis Excluding MR3 from the Migration Readiness Composite

Relationship	Main Model β	Sensitivity Model β	Sensitivity p-value	Main Model f^2	Sensitivity Model f^2
PUSH → MR	0.117	0.115	0.009	0.015	0.014
DPO → MR	−0.128	−0.134	<0.001	0.021	0.023
PEP → MR	0.272	0.259	<0.001	0.065	0.058
QLP → MR	0.221	0.223	<0.001	0.043	0.043
PB → MR	−0.187	−0.192	<0.001	0.051	0.052
SB → MR	−0.175	−0.169	<0.001	0.044	0.041
**R^2 MR**	**0.392**	**0.383**	—	—	—

Note: The sensitivity model excluded MR3 (perceived professional preparedness) while retaining MR1, MR2, and MR4 as formative indicators of Migration Readiness. All other measurement specifications, structural relationships, estimation settings, and the analytical sample remained unchanged.

Supplementary Table S2. Measurement Properties of the Migration Readiness Composite in the Sensitivity Model

Indicator	Outer VIF	Outer Weight	t-value	p-value	Outer Loading
MR1	2.007	0.288	3.073	0.002	0.836
MR2	2.078	0.397	4.699	<0.001	0.882
MR4	2.007	0.455	4.785	<0.001	0.897

Note: MR3 was excluded from the sensitivity specification. All remaining MR indicators showed statistically significant outer weights and acceptable collinearity, supporting the stability of the reduced formative Migration Readiness composite.

Supplementary Table S3. Comparison of Specific Indirect Effects Between the Main and Sensitivity Models

Specific Indirect Effect	Main Model β	Sensitivity Model β	Sensitivity Result
PEP → QLP → MR	0.116	0.117	Significant
PUSH → PEP → MR	0.112	0.107	Significant
PUSH → QLP → MR	0.038	0.038	Significant
PUSH → PEP → QLP → MR	0.048	0.048	Significant
DPO → PEP → MR	−0.048	−0.046	Significant
DPO → QLP → MR	−0.029	−0.029	Significant
DPO → PEP → QLP → MR	−0.021	−0.021	Significant
PUSH → DPO → MR	0.050	0.052	Significant
PUSH → DPO → PEP → MR	0.019	0.018	Significant
PUSH → DPO → QLP → MR	0.011	0.011	Significant
PUSH → DPO → PEP → QLP → MR	0.008	0.008	Significant

Note: All specific indirect associations retained their statistical significance after MR3 was excluded from the formative Migration Readiness composite. The magnitude and direction of the indirect associations remained substantively comparable with those observed in the main model.
